# Relationship between Transmission Intensity and Incidence of Dengue Hemorrhagic Fever in Thailand

**DOI:** 10.1371/journal.pntd.0000263

**Published:** 2008-07-16

**Authors:** Suwich Thammapalo, Yoshiro Nagao, Wataru Sakamoto, Seeviga Saengtharatip, Masaaki Tsujitani, Yasuhide Nakamura, Paul G. Coleman, Clive Davies

**Affiliations:** 1 Bureau of Vector Borne Disease, Department of Disease Control, Ministry of Public Health, Nonthaburi, Thailand; 2 Osaka University Graduate School of Medicine, Suita, Osaka, Japan; 3 London School of Hygiene and Tropical Medicine, London, United Kingdom; 4 Osaka University Graduate School of Engineering, Toyonaka, Osaka, Japan; 5 Osaka Electro-Communication University, Neyagawa, Osaka, Japan; 6 Osaka University Graduate School of Human Sciences, Suita, Osaka, Japan; University of Michigan, United States of America

## Abstract

**Background:**

Dengue is the most prevalent mosquito-borne virus, and potentially fatal dengue hemorrhagic fever (DHF) occurs mainly in secondary infections. It recently was hypothesized that, due to the presence of cross-immunity, the relationship between the incidence of DHF and transmission intensity may be negative at areas of intense transmission. We tested this hypothesis empirically, using vector abundance as a surrogate of transmission intensity.

**Methodology/Principal Findings:**

House Index (HI), which is defined as the percentage of households infested with vector larvae/pupae, was obtained from surveys conducted on one million houses in Thailand, between 2002 and 2004. First, the utility of HI as a surrogate of transmission intensity was confirmed because HI was correlated negatively with mean age of DHF in the population. Next, the relationship between DHF incidence and HI was investigated. DHF incidence increased only up to an HI of about 30, but declined thereafter. Reduction of HI from the currently maximal level to 30 would increase the incidence by more than 40%. Simulations, which implemented a recently proposed model for cross-immunity, generated results that resembled actual epidemiological data. It was predicted that cross-immunity generates a wide variation in incidence, thereby obscuring the relationship between incidence and transmission intensity. The relationship would become obvious only if data collected over a long duration (e.g., >10 years) was averaged.

**Conclusion:**

The negative relationship between DHF incidence and dengue transmission intensity implies that in regions of intense transmission, insufficient reduction of vector abundance may increase long-term DHF incidence. Further studies of a duration much longer than the present study, are warranted.

## Author Summary

An infection with dengue virus may lead to dengue hemorrhagic fever (DHF), a dangerous illness. There is no approved vaccine for this most prevalent mosquito-borne virus, which infects tens of millions (or more) people annually. Therefore, health authorities have been putting an emphasis on reduction of vector mosquitoes, genus *Aedes*. However, a new mathematical hypothesis predicted, quite paradoxically, that reducing *Aedes* mosquitoes in highly endemic countries may “increase” the incidence of DHF. To test this hypothesis based upon actual data, we compared DHF incidence collected from each of 1,000 districts in Thailand to data of *Aedes* abundance, which was obtained by surveying one million households. This analysis showed that reducing *Aedes* abundance from the highest level in Thailand to a moderate level would increase the incidence by more than 40%. In addition, we developed computer simulation software based upon the above hypothesis. The simulation predicted that epidemiological studies should be continued for a very long duration, preferably over a decade, to clearly detect such a paradoxical relationship between *Aedes* abundance and incidence of DHF. Such long-term studies are necessary, especially because tremendous efforts and resources have been (and perhaps will be) spent on combating *Aedes*.

## Introduction

Dengue is the most prevalent vector-borne viral disease, the distribution of which has been expanding continually [1]. Dengue virus is transmitted by *Aedes* mosquitoes [2]–[4], which breed predominantly in water-holding containers within human habitats. Infections with dengue virus may manifest as dengue fever (DF), or the potentially more fatal dengue hemorrhagic fever (DHF). There are four serotypes of dengue virus, among which transient cross-protection exists [5]. Dengue virus is unique in that viral amplification in a primate host is enhanced dramatically in the presence of pre-existing immunity to a heterogeneous dengue serotype(s). This phenomenon, called antibody-dependent enhancement (ADE), had been reported initially in other arthropod-borne virus infections [6],[7]. In terms of dengue, ADE was demonstrated both by *in vitro* [8] and animal experiments [9]. Subsequently, pre-existing hetero-serotypic antibodies were shown to be associated with elevated risk for development of DHF [10].

Although the periodicity of highly oscillatory DHF outbreaks has been under intensive study [11],[12], determinants of the absolute magnitude of DHF incidence remain poorly understood. It would be understandable if the incidence of DF or DHF were affected positively by transmission intensity (measured either as force of infection or basic reproductive number). However, this intuitive thinking may be too naive in terms of dengue illness. As an example, increases in DF observed in Singapore were thought to be due to insufficient vector reduction [13],[14]. This paradox may be explained as follows, at least to some extent, by the age-dependent manifestation of DF [15],[16]. Under more intense transmission, infections occur at earlier ages [17]. Primary infections of younger children often result in no symptoms or mild illness [16],[18]. As a result, many infections do not manifest as clinical DF under high transmission intensity, and consequently, the incidence of DF decreases. This state of low incidence of clinical illness under intense transmission is known as “endemic stability” [15].

In contrast to DF, children seemed to be more prone to manifest DHF than are adults [19]–[21]. However, these studies, which did not fully consider the immunological status of the hosts, cannot be compared easily. This lack of reliable information about age-dependency in the manifestation of DHF has made it difficult to predict whether endemic stability occurs for DHF. On the other hand, a mathematical model recently predicted that, due to the presence of transient cross-serotype immunity, the incidence of DHF and transmission intensity will be correlated negatively at high transmission intensities [22]. This model hypothesized that a cross-protected individual will be seroconverted to an infecting viral serotype, while he/she is protected from manifesting severe illness. Under this assumption, which is consistent with results from experiments on monkeys [23], the individual would acquire immunity to nearly all serotypes while being cross-protected from clinical illness, at very intense transmission. As a result, the incidence of DHF could be correlated negatively to transmission intensity at areas of intense transmission, while the correlation is positive only at low levels of transmission. In the present study, such a complex correlation structure mixed with positive and negative correlations will be called “non-monotonic”, hereafter. To the contrary, correlation structure, which is simply either positive or negative, is referred to as “monotonic”.

The present study aims to provide an empirical example of this non-monotonic relationship between the incidence of DHF and transmission intensity, with transmission intensity represented by vector abundance. Vector abundance is one of the major determinants for transmission intensity of a vector-borne disease [24]. Accordingly, the WHO recommends that vector abundance be quantified in regions highly infested with *Aedes* through breeding site surveys and/or adult mosquito collections [25]. In developing countries, breeding site survey is preferred over mosquito collection, since the former is less labor-intensive. These surveys measure the number of houses or water containers infested by *Aedes* larvae/pupae through standard larval indices, such as House index (HI) and Breteau index (BI). HI is defined as the percentage of all surveyed houses in which *Aedes* larvae or pupae are present, while BI represents the number of infested containers in 100 houses. BI was shown to be relatively sensitive in predicting transmission [26]. Since HI and BI are strongly positively correlated [26], HI also may reflect transmission intensity to some extent. Although the absolute number of pupae is thought to reflect transmission intensity more directly than do larval indices [27]–[29], Southeast Asian households often possess many large water containers [30], that are irregularly shaped and partially sealed, making it difficult to obtain precise estimates of the absolute number of pupae. For this reason, absolute pupal counts have not been used in large-scale surveys in Thailand.

Here, we describe the empirical relationship between DHF incidence and transmission intensity, as represented by HI. The age-specific structure of this relationship also was characterized to support findings obtained for the entire population. The epidemiological characteristics of DHF were compared with predictions made by simulation of an individual-based model based upon the above mentioned mathematical modelling study. Our findings have major implications for future epidemiological surveys and dengue control programs.

## Methods

### *Aedes* survey

In Thailand, the highest incidence of DHF occurs between June and August. Hence, entomological surveys mainly are conducted in the pre-epidemic season (*e.g.*, April), with the assumption that vector abundance in this season will serve as an indicator of disease incidence later in the year. Between 2002 and 2004, a large-scale national *Aedes* survey was conducted in all 914 districts of Thailand. The survey was intended partly for community education and was implemented by investigators dispatched from 302 vector control units and community volunteers, under the supervision of the five regional vector-borne disease control offices. The administrative central village or municipality of each sub-district was surveyed because of their accessibility. A total of 40 houses were visited in each village/municipality. Prior appointments with the residents were not made, so that the residents did not clean their houses in advance. This design, taken with incomplete house registry in rural areas, made genuine randomization impossible. Surveys were conducted in April of each year, with 9,483 villages surveyed in 2002, 9,763 in 2003, and 7,482 in 2004. The HI values were averaged for each district for comparison with district-level DHF data. The average population of a district in Thailand is 67,500.

### Epidemiological data

The Bureau of Epidemiology, Ministry of Public Health, provided the annual number of cases of DHF (including Dengue Shock Syndrome) in nine age categories (0–4, 5–9, 10–14, 15–24, 25–34, 35–44, 45–54, 55–64, ≥65 years) for each district, for the years between 1994 and 2004. Age-stratified population data, based upon five yearly censuses/surveys and yearly projections, were obtained from the National Statistics Office of Thailand (http://www.nso.th.go) to calculate DHF incidence. The incidence of DHF in an entire district population was adjusted to the national age-population structure of 2000 by using the Direct Method, to eliminate possible interference by the heterogeneity in demographic structure. The mean age of DHF cases was calculated as the average of the mid-point of the different age categories (2.5, 7.5, 12.5, 20, 30, 40, 50, 60, and 75 years) weighted by the number of cases in each category.

### Statistical methods

Subsequent statistical analyses were performed using R 2.6.2 and Stata 9.2. We used non-parametric statistical methods, Spearman's rank correlation analysis and the generalized additive model (GAM), so that analyses did not have to assume any fixed distribution *a priori*. Akaike's Information Criteria (AIC) inversely represented the goodness of fit, or predictability, for a regression model obtained from GAM [31]. Deviance around the prediction also was presented, although this measurement is not adjusted for degree of freedom (*df*) used in a regression model.

### Analysis of relationship between DHF mean age and HI

To ensure that HI could be used as a reliable surrogate of transmission intensity, we compared the mean age of DHF cases to HI using rank correlation analysis. A high mean age of DHF cases was used as an indicator of low transmission intensity, because the mean age of infected individuals generally is negatively correlated with the transmission intensity of an acute infectious disease [17]. Since each district was surveyed three times (2002, 2003, and 2004), the possible bias from this repeated measurement was adjusted by simply aggregating records from three years for each district. Among the all 914 districts, this analysis incorporated 909 districts that reported at least one case of DHF between 2002 and 2004.

### Analysis of the relationship between DHF incidence and HI

We examined the quantitative relationship between incidence of DHF and HI using GAM. Logarithm was used as the link function. First, we tested this relationship by incorporating only HI as the independent variable (univariate analysis). Then, we adjusted for possible confounding by socioeconomic and climatic variables. Socioeconomic factors may affect reported incidence in diverse fashions. For example, incidence may be biased by (a) the prevalence of health offices, which are responsible for DHF case reporting in each district. Abundance of breeding places is affected by local water storage practices (reviewed by [32]). Our analysis incorporated the following socioeconomic factors that were reported to be associated with dengue transmission intensity [33]: (b) per capita number of public large water wells, (c) that of public small wells, (d) that of private small wells, (e) annual birth rate per 1,000 individuals, (f) proportion of households owning land, and (g) proportion of villages in which high schools are present. These seven socioeconomic variables (a–f), censused every other year, were obtained from the Information Processing Centre of Thammasat University, Bangkok, and were interpolated linearly to the intervening years. On the other hand, dengue transmission intensity is influenced by climatic factors as well. Temperature affects critically the rate of viral amplification in mosquitoes [34]. In addition, extremely high or low temperatures are rate-determining factors for the growth and survival of mosquitoes [35]. Atmospheric vapor pressure is known to affect dengue transmission [36]. Aridity, which is likely to reflect the scarcity of underground water, may be associated with increased use of household water containers. To adjust for these possible confounders, the following climatic variables were obtained from the University Cooperation for Atmospheric Research [37]: (a) temperature averaged between January and February, the coolest months in Thailand (“winter temperature”, °C), (b) temperature averaged between April and May, the hottest months (“summer temperature”, °C), (c) average vapor pressure (AVP, hPa), and (d) average pan evapo-transpiration (APET, mm/day). These climatic variables were obtained from 89 weather stations in Thailand and its adjacent countries, averaged for each year, and interpolated to the geographic centroid of each district by using Inverse Distance Weighting method. We confirmed that multiple interpolation methods generated comparative results, perhaps because these weather stations constituted a sufficiently exhaustive dataset [38].

Collectively, these socioeconomic/climatic variables were averaged for the period for which the dependent variable, incidence, was averaged. We enrolled districts from which socioeconomic and climatic variables have been available from 1994 to 2004. Consequently, 785 districts were enrolled. This dataset (incidence linked with covariates) is available on request from the corresponding author. Multivariate analyses were conducted using the following procedure. First, HI and all socioeconomic/climatic factors were incorporated as independent variables, with *df* of each variable set to 2. Next, independent variables that remained significant (*P*<0.05) in a stepwise elimination procedure were selected, generating the “smallest regression model for *df* = 2”. Finally, *df* of each of the remaining six variables was replaced with *df* = 3, generating 2^6^ combinations of *df*. Among these, the combination that exhibited the smallest AIC was adopted as the “final regression model”.

### Age-stratified analysis of DHF incidence and HI

The relationship between DHF incidence and HI was examined within different age classes for which original age categories were aggregated into the following three age classes: 0–4, 5–24, and ≥25 years. GAM was applied similarly to these age-class specific incidences.

### Simulation

We employed computer simulations to see whether (and to what extent) the observed epidemiological pattern could be explained based upon a theoretical framework. The assumption of the above mentioned mathematical model was expressed equivalently by an individual-based model (see Protocol S1, Section I). This model is summarized as follows. The cross-protective period was assumed to be of a fixed duration (“*C*” years). Inoculation by a virus, which occurred during this cross-protective period, does not develop into DHF, but induces seroconversion. As the cross-protective period expires, the individual is predisposed to the risk of manifesting DHF in a subsequent inoculation by a secondary (or later) serotype. An individual could manifest DHF after secondary, tertiary or quaternary infections.

In addition, this individual-based model can incorporate the age-dependency in the probability to manifest DHF (categorical parameter “*A*”, defined in Figure S1B in Protocol S1). Transmission intensity is represented by basic reproductive number (R_0_) of dengue virus. The present study parameterized simulations with the following three scenarios. (I) Cross-immunity scenario: the duration of cross-serotype protection (“*C*”) was set to two years, while the probability to manifest DHF was assumed to be independent of age (*A* = 0). We selected this duration of cross-immunity based upon the results of sensitivity analysis (see Protocol S1). (II) Age-dependency scenario: the probability to manifest DHF in secondary or later infections was assumed to increase in accordance with the age of the individual (*A* = 2), while no cross-immunity was assumed (*C* = 0). (III) Control scenario: no cross-immunity or age-dependency was assumed (*C* = 0, *A* = 0). R_0_ was selected by extrapolating the mean age of DHF obtained between 2002 and 2004 from each of the 785 districts, through the relationship between R_0_ and mean age of DHF (Figure S6 in Protocol S1). This set of R_0_ values was used as the input for all three scenarios. Each simulation was run for 150 years.

### Comparison of predictability between simulations and actual incidence

At different durations for averaging (*W*), the goodness of fit was compared between the statistical models that explained the incidence in simulations versus those that explained the actual incidence. Incidences of DHF generated from simulations were averaged from the last *W* years [*W* = 3, 4 … 40] (for example, 148th, 149th and 150th years were averaged for *W* = 3). Subsequently, the averaged incidence was regressed against R_0_ using GAM.

On the other hand, actual incidences were averaged for the recent *W* years [*W* = 3, 4 … 11] (for example, *W* = 3 corresponds to 2002–2004; *W* = 11 corresponds to 1994–2004). Then, the averaged actual incidence was regressed against HI obtained from the 2002–2004 survey, and socioeconomic/climatic variables averaged for the recent *W* years.

## Results

### Relationship between mean age of DHF and HI

**Figure 1:**
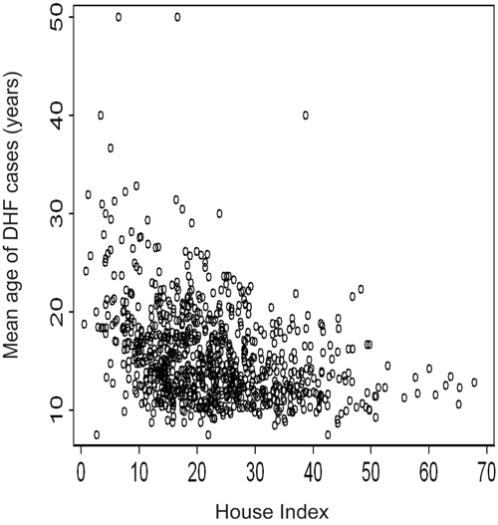
Relationship between mean age of Dengue Hemorrhagic Fever (DHF) cases and House Index in Thailand, averaged between 2002–2004. Each point corresponds to one district in this and subsequent figures.

The national-level mean age of DHF cases was 16 years during 2002 to 2004. The mean HI recorded each April during 2002 to 2004 was 23. As shown in Figure 1, the mean age was negatively correlated with HI at the district level (Spearman's R = −0.35, *P*<0.0001, N = 909).

### Relationship between DHF incidence and HI

**Figure 2:**
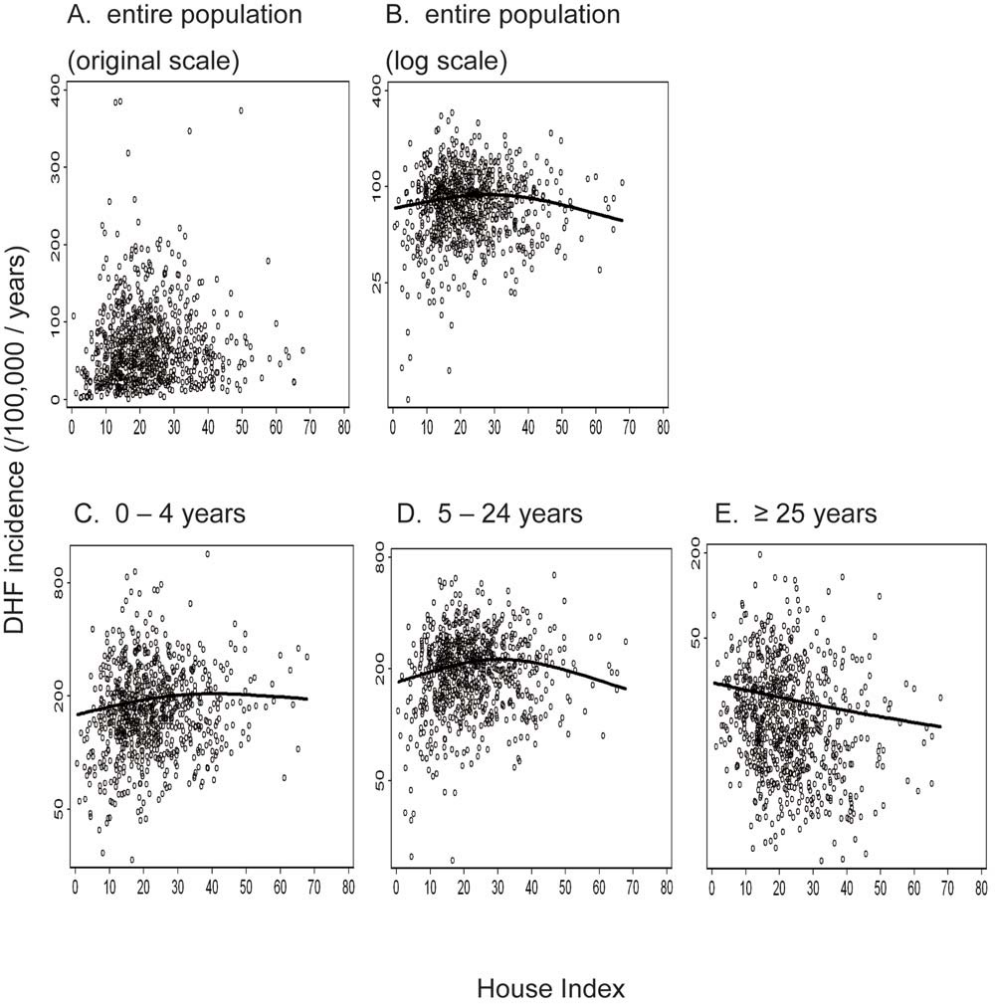
Relationship between DHF incidence and House Index, observed between 2002 and 2004 in Thailand. The annual incidence of DHF (per 100,000 individuals) averaged between 2002 and 2004 is plotted against House Index in the entire population (A, B), at 0–4 years (C), 5–24 years (D), and ≥25 years (E). Y-axis is original scale (A) or log scale (B–E). The lines correspond to the univariate regression model presented in Table 1 for the entire population, and in Table 2 for the age-stratified population.

**Figure 3:**
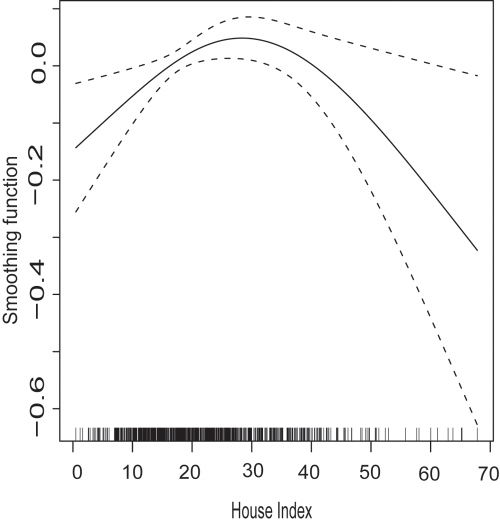
Non-linear responses of the log incidence of DHF to House Index, revealed by univariate analysis of generalized additive model (GAM). The smoothing function obtained from GAM analysis, which regressed the log of DHF incidence reported between 2002 and 2004 against House Index, is presented as the solid line. Dashed line represents the 95% confidence interval, while the spikes on the horizontal axis represent the frequency of data points (or districts) in this and the next figure.

During 2002 to 2004, the annual DHF incidence was 83 per 100,000 individuals. HI showed a statistically significant contribution to the log incidence of DHF, both in univariate and multivariate regression models (Table 1). Univariate analysis of GAM revealed that the correlation between HI and incidence was positive below about HI = 30, while the correlation was negative above this HI value (Figure 2B; Figure 3). As HI decreases from 70 to 30, for example, the log incidence would increase by 0.35 (Figure 3), which is equivalent to an increase of 40% in incidence, since exp (0.35) = 1.4.

**Table 1:** Contribution of vector abundance and socioeconomic/climatic factors in explaining log incidence of Dengue Hemorrhagic Fever averaged between 2002 and 2004, revealed by generalized additive model (GAM).

N = 785			
1. univariate regression model (*df* ^†^ = 2)			
Variable		*P* values	
House Index	0.006		
Deviance	2,092,809		
AIC ^‡^	8,428		
2. multivariate regression model			
	full regression model for *df* = 2	smallest regression model for *df* = 2	final regression model with mixed *df*
variables ^§^		*P* values	
House Index	0.028	0.035	0.031 (*df* = 2)
APET	<0.001	<0.001	<0.001 (*df* = 2)
winter temperature	<0.001	<0.001	<0.001 (*df* = 2)
summer temperature	0.003	<0.001	<0.001 (*df* = 2)
public large wells	0.004	<0.001	<0.001 (*df* = 3)
birth rate	<0.001	<0.001	<0.001 (*df* = 2)
AVP	0.651		
health stations	0.468		
high schools	0.301		
public small wells	0.846		
private small wells	0.401		
land ownership	0.714		
Deviance	1,558,853	1,572,931	1,566,715
AIC	8,240	8,224	8,223

^†^ df: degree of freedom; ^‡^ Akaike's Information Criterion; ^§^ AVP: average vapor pressure (hPa); APET: average pan evapo-transpiration (mm/day). Socioeconomic variables are defined in the text.

**Figure 4:**
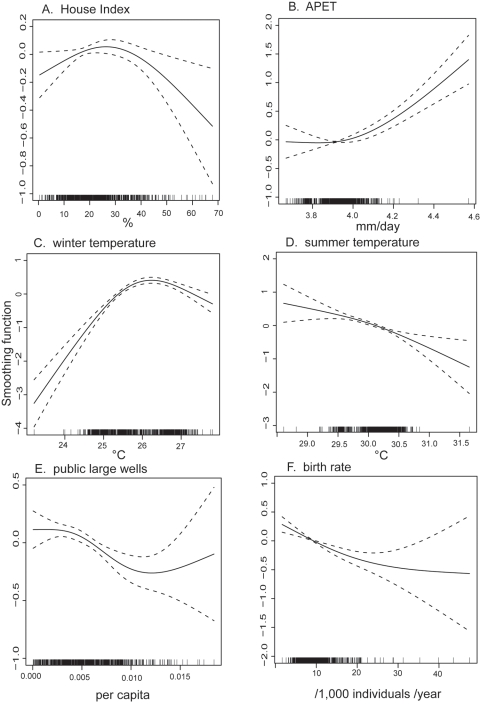
Non-linear responses of the log incidence of DHF to House Index and socioeconomic/climatic variables revealed by multivarite analysis of GAM. The final regression model obtained from the multivariate GAM analysis (Table 1) is presented. Log incidence of DHF reported between 2002 to 2004 was regressed against House Index and socioeconomic/variables. The per capital number of public large wells was assigned with *df* = 3, while other covariates were assigned with *df* = 2.

In multivariate analysis, the following six variables remained in the final regression model (Table 1; Figure 4): HI, winter temperature, summer temperature, APET, public large wells, and birth rate. The best predictability (or lowest AIC) was achieved by the final regression model which assigned *df* = 3 only to public large wells, and *df* = 2 to other covariates. Multivariate analysis estimated that, as HI decreases from 70 to 30, log incidence would increase by 0.6 (Figure 4A), which corresponds to an increase of 80%.

**Figure 5:**
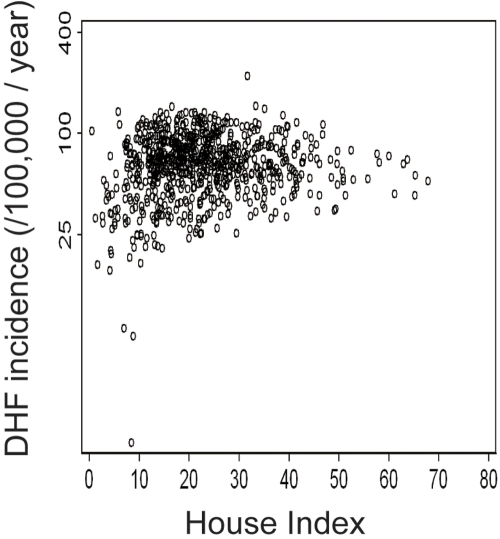
Prediction of DHF incidence based upon House Index and socioeconomic/climatic variables. Incidence of DHF was predicted based upon the final regression model (Table 1) and plotted against House Index. Y-axis is log scale.

Although incorporation of socioeconomic/variables improved the goodness of fit, this multivariate predictive model still failed to reproduce the very wide variation in the observed incidence (compare Figure 2B vs Figure 5).

### Age-specific relationship between DHF incidence and HI

Further analysis of the age-specific associations between incidence of DHF and HI was conducted, as shown in Table 2. Univariate analysis revealed that incidence and HI were positively correlated in the youngest age class (Figure 2C); whereas, DHF incidence and HI were negatively correlated in the oldest age class (Figure 2E). A non-monotonic relationship between DHF incidence and HI was detected within the intermediate age class (Figure 2D). When the socioeconomic/climatic variables were incorporated, the statistical significance of positive correlation among the youngest age class diminished (Table 2).

**Table 2:** Relationship between vector abundance and incidence of DHF stratified for age classes, revealed by GAM.

N = 785			
Age class	0–4 years	5–24 years	≥25 years
1. univariate regression model (*df* ^†^ = 2)			
variable		*P* values	
House Index	0.044	0.001	0.008
			
Deviance	5,592,654	12,851,371	290,947
AIC ^‡^	9,200	9,852	6,879
2. multivariate regression model (final regression model with mixed *df*)			
variables ^§^		*P* values	
House Index		0.031 (*df* = 3)	0.002 (*df* = 2)
APET	<0.001 (*df* = 3)	<0.001 (*df* = 2)	0.006 (*df* = 2)
winter temperature	<0.001 (*df* = 2)	<0.001 (*df* = 2)	0.003 (*df* = 2)
summer temperature	<0.001 (*df* = 2)	<0.001 (*df* = 2)	0.005 (*df* = 2)
public large wells		0.002 (*df* = 3)	<0.001 (*df* = 2)
birth rate		<0.001 (*df* = 2)	
AVP			<0.001 (*df* = 3)
health stations			
high schools			0.008 (*df* = 3)
public small wells			
private small wells			<0.001 (*df* = 2)
land ownership	0.001 (*df* = 2)		<0.001 (*df* = 3)
Deviance	4,592,236	10,128,066	165,907
AIC	9,058	9,689	6,476

^†^ df: degree of freedom; ^‡^ Akaike's Information Criterion; ^§^ AVP: average vapor pressure (hPa); APET: average pan evapo-transpiration (mm/day). Socioeconomic variables are defined in the text.

### Simulation

**Figure 6:**
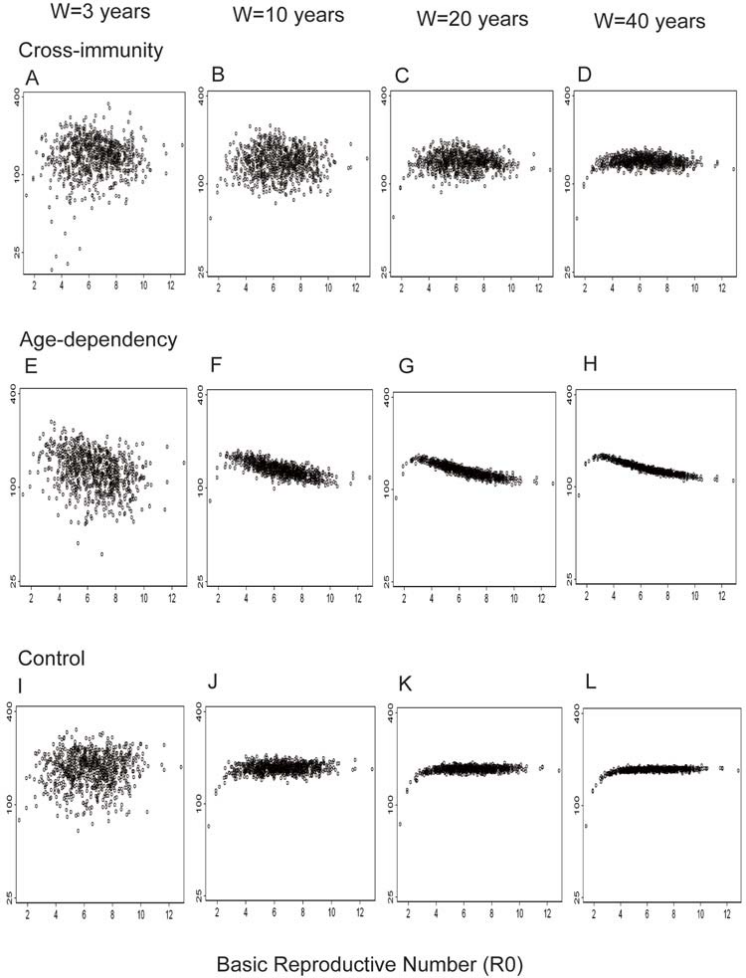
Relationship between DHF incidence and transmission intensity predicted by simulations. Incidence of DHF generated by simulations are plotted over Basic Reproductive Number (R_0_). The used scenarios are: two-year cross-immunity (A–D); age-dependency which assumed a higher probability of manifesting DHF in the older individuals (E–H); and control scenario without cross-immunity or age-dependency (I–L). Incidences in the last “*W*” years in each simulation were averaged, where *W* = 3 years (A, E, I), 10 years (B, F, J), 20 years (C, G, K), or 40 years (D, H, L). Y-axis is log scale.

As shown in Figure 6, averaging only the last three years of each simulation resulted in a negligibly detectable relationship between DHF incidence and R_0_, which greatly resembled the empirical relationship (compare with Figure 2B). As the window for averaging increased, the relationship generally became more apparent. The incidences generated by simulations with cross-immunity were much more dispersed than those generated by other simulations, at any window lengths.

**Figure 7:**
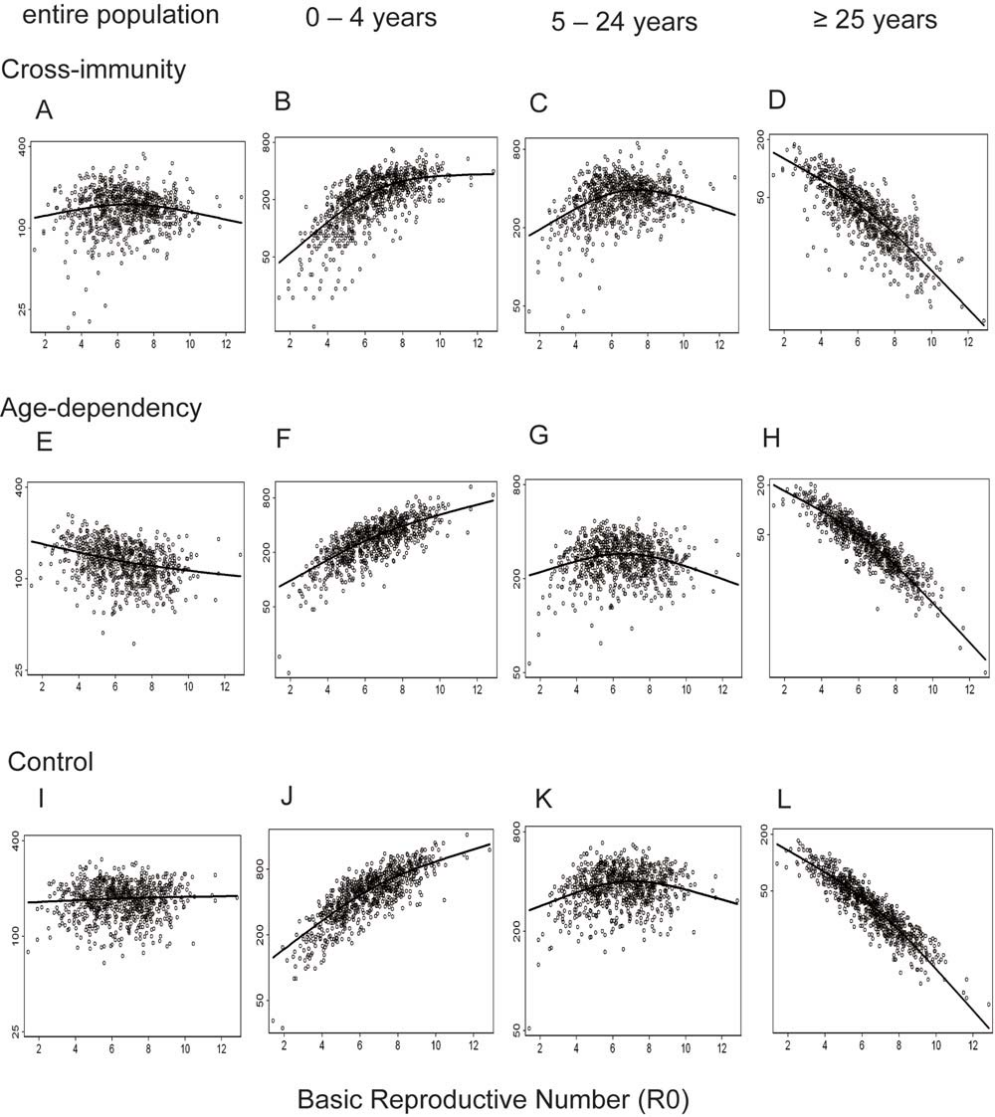
Age-specific relationship between DHF incidence and transmission intensity predicted by simulations. Age-stratified incidence of DHF in the last three years from each simulation were averaged. The parameters inputted to simulations were: two-year cross-immunity (A–D); age-dependency which assumes a higher probability of manifesting DHF in older individuals (E–H); control scenario without cross-immunity or age-dependency (I–L). DHF incidence presented for the entire population (A, E, I) or stratified into 0–4 years (B, F, J), 5–24 years (C, G, K), and ≥25 years (D, H, L). Y-axis is log scale.

GAM was applied to examine the relationship between incidences generated by simulations and R_0_ (Figure 7). As a result, GAM detected a non-monotonic relationship in the simulations with cross-immunity (Figure 7A), a negative relationship in those with age-dependency (Figure 7E), and a slightly positive relationship in the control simulations (Figure 7I). Age-stratification of the simulation results generated a similar trend in the empirical data, regardless of the presence of cross-immunity or age-dependency (Figure 7B–D, F–H, J–L). That is, a positive correlation was observed between DHF incidence and transmission intensity in the younger population, and a negative correlation was present in the older population.

### Comparison of predictability

**Figure 8:**
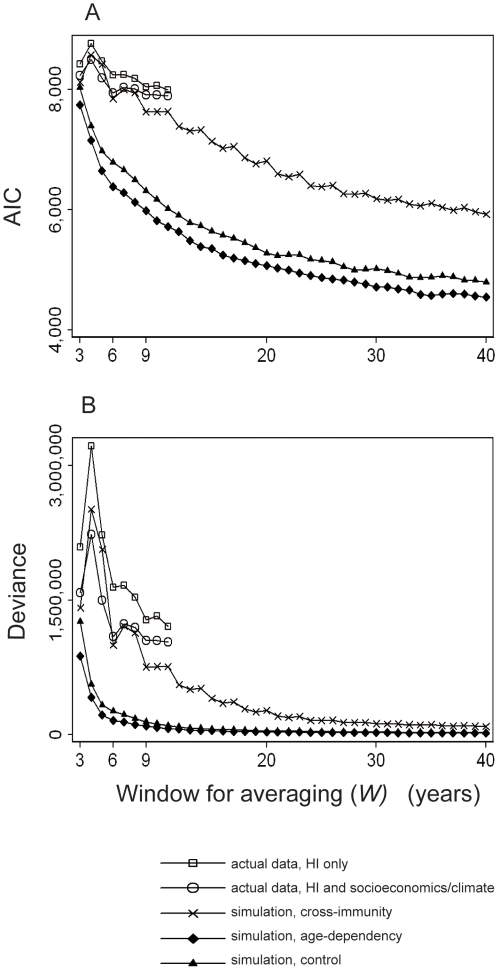
Goodness of fit in predicting the incidence of DHF obtained either from actual epidemiological data, or from simulations. The goodness of fit in the regression models for incidence of DHF is plotted over the window length (“*W*”) with which the incidence was averaged. For actual epidemiological data, the most recent *W* years descending from 2004 were averaged. For simulations, the last *W* years in each simulation were averaged. Actual incidence was regressed against HI only (square), or against HI and socioeconomic variables (circle) (Table 3), while incidence in simulations were regressed against R_0_ with *df* = 2 assigned. The goodness of fit as inversely measured by Akaike's Information Criterion (AIC) (A), and the deviance (B) were obtained from GAM models. The scenario parameters for simulations were: cross-immunity (X); age-dependency (diamond); or control scenario without cross-immunity or age-dependency (triangle).

**Figure 9:**
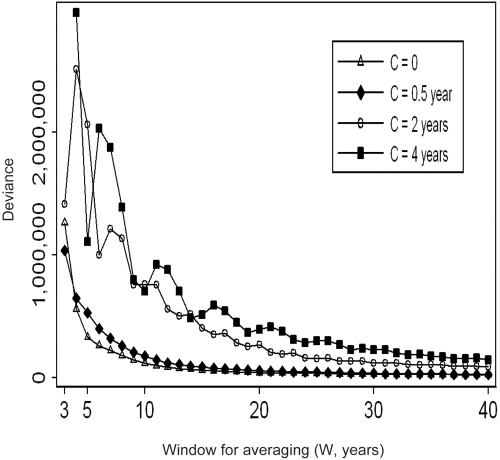
Responses of predictability of DHF incidence to the window length for averaging, at diverse durations of cross-immunity. The predictability of DHF incidence was inversely measured by deviance and plotted to *W* (see the legend for Figure 8). Simulations with diverse durations of cross-immunity examined in the sensitivity analysis are compared. The results for cross-immunity (*C*) of 0, 0.5, 2 and 4 years are presented.

The goodness of fit in predicting incidence by R_0_ showed remarkable differences between simulation with cross-immunity and those without cross-immunity (Figure 8). The predictability was much worse in simulation with cross-immunity than in other simulations. In addition, the response to the window length was more complex in the presence of cross-immunity than in other simulations. That is, in simulations without cross-immunity, the predictabilities improved continuously as *W* increased. In contrast, the predictability in the presence of cross-immunity deteriorated as the window for averaging increased from *W* = 3 to *W* = 4, then improved up to *W* = 6. With a small setback at *W* = 7, it improved again thereafter. Such a complex response of predictability to *W* was reproduced at diverse durations of cross-immunity (Figure 9), which were sufficiently long to generate dominant supra-annual periodicities (see Section II and Figure S7 in Protocol S1).

The goodness of fit in predicting actual incidence, either by HI only or by HI and covariates, showed a similarly complex response to *W* (Table 3). The predictability attained solely by HI was much inferior to that attained in any simulations (Figure 8). However, the predictability using the multivariate regression model was as good as that in simulations with cross-immunity, up to *W* = 8.

**Table 3:** Contribution of vector abundance and socioeconomic/climatic factors in explaining log incidence of DHF averaged over diverse durations, revealed by GAM.

N = 785									
window for averaing (years)	3	4	5	6	7	8	9	10	11
1. univariate regression model (*df* ^†^ = 2)									
variable	level of *P* values								
House Index	**	**	**	*	*	*	**	**	*
AIC ^‡^	8,428	8,767	8,477	8,239	8,250	8,183	8,040	8,069	7,993
2. multivariate regression model (final regression model with mixed *df*)									
variables ^§^	level of *P* values								
House Index	*	*	*	*	*	*	*		
APET	***	***	***	***	***	***	**	*	
winter temperature	***	***	***	***	***	***	***	***	***
summer temperature	***	**			***	*	*	***	*
public large wells	***	**	**	**	*				
birth rate	***	***	***	***	***	***	***	***	***
AIC	8,223	8,497	8,180	7,932	8,037	8,011	7,908	7,913	7,905

^†^ *df*: degree of freedom; ^‡^ Akaike's Information Criterion; ^§^ APET: average pan evapo-transpiration (mm/day). Socioeconomic variables are defined in the text. Variable “public large wells” was assigned *df* = 3. All other independent variables were assigned *df* = 2. ^*^: *P*<0.05, ^**^: *P*<0.01, ^***^: *P*<0.001.

## Discussion

Our analysis demonstrates that HI is a reliable indicator of transmission intensity, at least at the district level. The usefulness of HI is evident by its highly significant, inverse relationship to mean age, otherwise equivalent to a positive correlation between HI and transmission intensity. Our findings are consistent with observations from Singapore, where an increase in the mean age of patients with dengue infection was preceded by a substantial reduction in HI [13],[14].

Analysis of DHF incidence among the entire Thai population revealed that incidence rose up to HI of about 30 and gradually declined thereafter. This non-monotonic relationship appears to be consistent with a state of endemic stability. However, the age-dependency in the probability to manifest DHF may not simply satisfy the condition for endemic stability, because DHF occurs more frequently in children than in adults. On the other hand, cross-immunity explains not only this non-monotonic relationship (Figure S2 in Protocol S1), but also the wide variation in incidence of DHF, as well as its complex response to the duration of window for averaging (Figure S8 in Protocol S1). Of note, the regression model comprised of HI and socioeconomic/climatic variables predicted actual incidence to the same goodness of fit, with which R_0_ predicted incidence from simulations with cross-immunity (Figure 8). This finding may support the validity of the multivariate regression model, and that of our assumption for cross-immunity, simultaneously.

Stratification of data according to age revealed a positive association between DHF incidence and HI among the youngest population. In contrast, a negative association was observed in the oldest population. These contrasting correlations may be explained as follows. Under low transmission intensity, the majority of individuals in the youngest age class do not possess antibodies against any serotype and are relatively resistant to DHF. As the transmission intensity increases, a larger number of individuals in this age class possess antibodies to only one serotype, making them predisposed to DHF. Therefore, the correlation between DHF incidence and transmission intensity becomes positive in the youngest age class, as observed here. In contrast, when transmission intensity is low, many in the oldest age class possess antibodies against only one serotype and are predisposed to DHF. As transmission intensity increases, more members of this age class possess antibodies against almost all serotypes, conferring resistance to DHF. Importantly, these age-stratified relationships could be reproduced by simulations of any scenarios examined. Therefore, this analysis did not differentiate whether cross-immunity or age-dependency determined the epidemiological characteristics of DHF.

The negative response of incidence to transmission intensity at areas of intense transmission has important public health implications, regardless of its underlying mechanism. The incidence of DHF is affected by the dominant virus serotype, which shifts from period to period [39],[40]. In addition, HI measured in one country cannot be compared with HI in another country. Since our analysis was based on a single three-year period in one country, the stability of our estimated HI value at the maximum (“turning”) point should be treated with some caution. However, with these caveats, our results indicate that insufficient reduction of vector abundance in highly endemic areas could result in an increased incidence of DHF. As the HI decreases from the current highest level in Thailand, the incidence of DHF could increase by more than 40%. Any medical/public-health intervention that causes a foreseeable increase of illness should be subject to ethical discussion.

Theoretically, sufficiently radical reduction of vector mosquitoes can achieve a decrease of the entire incidence of DHF. However, it is unclear whether such radical vector control is possible at a nation-wide scale in developing countries. Instead, reduction of the vector population may become stagnant as the vector abundance decreases. Furthermore, even substantial vector reduction (for example, from HI = 60 to10) would not necessarily decrease the final incidence (extrapolate the HI values to incidence in Figure 3 and Figure 4A), but would result most likely in a greater number of DHF cases accumulated over the course of time. This calculation suggests that it is extremely difficult for vector control alone to achieve the ultimate goal of control program– reduction of incidence.

## Supporting Information

Protocol S1 (0.25 MB DOC)

Table S1 Definition and values of scenario parameters assigned to each simulation of Dengue Hemorrhagic Fever (DHF)
(0.04 MB DOC)

Figure S1 Individual-Based Model for Dengue Hemorrhagic Fever (DHF). A. Diagram of the transition between immunological states caused by infections with wild type virus. The transition between immunological states was a result of either viral inoculation (solid arrow) or expiration of time from the most recent inoculation (broken arrow). The serotype(s) that an individual has experienced is recorded as the existence of protective antibodies to this serotype(s). B. Age-dependent probability for a secondary infection to manifest as DHF in a DHF-predisposed individual. Four hypothetical possibilities of age-dependency are defined: no age-dependency (*A* = 0), higher probability in younger individuals (*A* = 1), higher probability in older individuals (*A* = 2), and complex age-dependent DHF manifestation (*A* = 3).
(0.74 MB TIF)

Figure S2 Relationship between DHF incidence and transmission intensity (R_0_) generated by cross-serotype immunity. Results from simulations, which assumed the cross-protective period (“*C*”) to be (A) 0 year, (B) 1 year, or (C) 2 years, are presented. No age-dependency was assumed for DHF manifestation (i.e., *A* = 0). Infection with four serotypes was required to confer life-long resistance to DHF (*L* = 4 serotypes). Transmission enhancement was not assumed (*E* = 1). Qualitatively similar results were obtained with *L* = 2 or 3 and with *E* = 2 or 20.
(0.21 MB TIF)

Figure S3 Relationship between DHF incidence and transmission intensity generated by age-dependent manifestation of DHF. The results of simulation are presented for four age-dependencies (*A*) of DHF manifestation. No cross-protection was assumed (i.e., *C* = 0 year). Infection with four serotypes was required to confer life-long immunity (*L* = 4 serotypes). Transmission enhancement was not assumed (*E* = 1). Qualitatively similar results were obtained with *L* = 2 or 3, and with *E* = 2 or 20.
(0.26 MB TIF)

Figure S4 Relationship between DHF incidence and transmission intensity generated by transmission enhancement. Transmission enhancement (*E*) during manifesting DHF was assumed to be 1 (no enhancement), 2 or 20. No age-dependency or cross-protection was assumed (i.e., *A* = 0, *C* = 0 year). Infection with four serotypes was required to confer life-long immunity (*L* = 4 serotypes). Qualitatively similar results were obtained with *L* = 2 or 3.
(0.18 MB TIF)

Figure S5 Temporal pattern of alternating serotypes in the presence of cross immunity and effects of a sudden drop in transmission intensity. Examples of serotype-specific incidence of DHF are presented. The last 40 years are presented.
(1.18 MB TIF)

Figure S6 Relationship between mean age of DHF cases and Dengue transmission intensity (R_0_) Mean age of DHF cases is plotted against transmission intensity (R_0_). The result for a parameter setting (*C* = 2 years, *L* = 4 serotypes, *A* = no age-dependency, *E* = 1) is presented. All other parameter combinations examined generated similarly negative correlations between mean age of DHF cases and R_0_.
(0.14 MB TIF)

Figure S7 Periodicity profile (periodogram) for incidence generated by simulations for Dengue Hemorrhagic Fever (DHF). Individual-based simulation for DHF (described in the accompanying manuscript) was executed for 150 years, from which monthly incidence for the last 40 years was analyzed by fast Fourier transform with Daniell smoothing (provided in R 2.6.2). Parameters for simulations are as follows: cross-immunity of 0.5 year (A–C), one year (D–F), two years (G–I), three years (J–L), and four years (M–O); age-dependency, which attributes a higher probability of manifesting DHF to the older population [defined as *A* = 2 in the accompanying manuscript] (P–R); control (i.e., no cross-immunity, no age-dependency) (S–U). Transmission intensity inputted to simulations were R_0_ = 3 (A, D, G, J, M, P, S), R_0_ = 6 (B, E, H, K, N, Q, T), or R_0_ = 12 (C, F, I, L, O, R, U). We executed each parameter setting in duplicate, and confirmed that the resulting periodograms were very similar. The highest spectrum intensity was presented as 1, for each parameter setting.
(0.87 MB TIF)

Figure S8 Standard deviation in the asynchronous sinusoidal incidences. One hundred sinusoidal curves, with asynchronous phases, were generated, to emulate the incidence of DHF. The sinusoidal incidence was averaged for diverse window lengths (“*W*”), and standard deviation was measured among these averaged incidences. A. Each sinusoidal time-series follows cycles of exactly one year (diamond) or three years (x). B. To add noise to the cycles, the cycle at each time-series was selected randomly between 0.8 and 1.2 years (diamond), or between 2.6 and 3.4 years (x).
(0.47 MB TIF)

Alternative Language Abstract S1 Translation of the Abstract into Japanese by Yoshiro Nagao
(0.09 MB PDF)
