# Supplementary material for: Relationship between Transmission Intensity and Incidence of Dengue Hemorrhagic Fever in Thailand
Source: PLoS Negl Trop Dis. 2008 Jul 16;2(7):e263. doi: 10.1371/journal.pntd.0000263 (PMC2442222; doi:10.1371/journal.pntd.0000263)
Supplement: Protocol S1 — (0.25 MB DOC) [file pntd.0000263.s001.doc]

**Protocol S1:**

**Supplementary File to**

**"Relationship between transmission intensity and incidence of Dengue Hemorrhagic Fever in Thailand"**

**Section I**

**Individual-Based Model for Dengue Hemorrhagic Fever**

This section describes the Individual-Based Model simulation software in detail. We did not invent a new disease model, rather, we translated a recently proposed mathematical model [1] into this individual-based model, so that the effects of cross-immunity and age-dependency can be compared on the same platform and because the original mathematical model did not take into account the effect of age. In addition, in this supplementary file, we examined the responses of the individual-based model to changes in input parameters in order to confirm that it faithfully reproduced the behavior of the original mathematical model.

**Model**

**Transmission intensity**

Since we treated four serotypes symmetrically, transmission intensity was expressed as the basic reproductive number (R_0_) [2,3]. The R_0_ of dengue is defined as the number of new viremic subjects produced from one viremic subject in a hypothetical population consisting only of naïve subjects. If an individual virus serotype possesses distinct affinities toward vector mosquitoes (most importantly, *Aedes aegypti*) or human hosts, expressing the transmission intensity with this single scalar variable may be inadequate. However, to our knowledge, there is no definitive information that supports using serotype-specific R_0_s.

**Assumptions regarding the immunology of DHF**

The model incorporated the following assumptions into immunological state transition diagram (Figure S1 A). To account for the immunology of DHF parsimoniously, the model software surmised two antigens/epitopes (or groups of antigens/epitopes): a protective antigen and an enhancing antigen. When an individual is inoculated with a virus, he/she acquires antibodies to the protective antigen of this serotype, as well as to the enhancing antigen shared by all serotypes. Based upon a previous report, we assumed that the protective antigen is serotype-specific, while the enhancing antigen is shared by serotypes [4]. The protective antigen of a serotype induces life-long sterile immunity to this serotype (i.e. direct protection), and transient immunity to other serotypes that lasts for "*C*" years from the most recent inoculation (i.e. cross-reactive protection). If an individual in the "cross-protected state" is inoculated with a virus, the individual will seroconvert, but will not develop DHF. *C* years after the most recent exposure to protective antigen, protective antibodies wane, and antibodies to the enhancing antigen predispose the individual to DHF. When this individual in the "DHF-predisposed state" is inoculated with a virus serotype against which he/she is not protected directly or cross-reactively, this individual will develop DHF according to a probability that is dependent upon age (mentioned later under **Assumptions regarding age-dependency**).

Viremia has been reported to be elevated during DHF [5]. A previous modeling study predicted that, if the transmissibility is elevated, four serotypes can co-exist without excluding each other [6,7]. Considering these studies, our model expressed the elevation of transmissibility in an individual manifesting DHF as a factor of "*E*", and assumed three possibilities of *E* = 1 (i.e., no transmissibility enhancement), 2 (i.e., double-fold increase in transmissibility), and 20.

We selected parameter values similar to those that were adopted elsewhere [1]. We generalized that infections with as many as “*L*” serotypes (*L* = 2, 3, or 4) are sufficient to confer life-long immunity against the manifestation of DHF resulting from inoculation with the remaining serotypes. There has been no agreement about whether "*L*" is 2 (as was advocated in ref. [8]) or more than 2 (as in ref. [9]). As a sensitivity analysis, we considered six possibilities for the length of cross-protection (*C* = 0, 0.5, 1, 2, 3, and 4 years). In our model, DHF also could occur during primary infection. The age-independent probability of DHF in primary infections was assigned 0.002, which corresponds to approximately one-twentieth of that in secondary infections [10,11]. We treated infants similarly to people of other ages. To our knowledge, no previous DHF models specifically addressed infants.

**Assumptions regarding age-dependency**

The age-dependent probability of manifesting DHF is a matter of debate. While there have been numerous reports suggesting that the younger population is at the highest risk for DHF [12-14], an even higher risk has been observed in the elderly [15-17]. Here, these two opposing scenarios were classified as “higher probability of DHF manifestation in younger individuals (*A* = 1)” and “higher probability of DHF manifestation in older individuals (*A* = 2)”, respectively. However, the age-dependency of DHF manifestation may not be so simple. The risk of DHF manifestation reportedly peaks at six years of age, with the risk being lower in younger children and in adults [18]. This age-dependency was incorporated as "complex age-dependency (*A* = 3)." As a control, we also allowed for the possibility that "no age-dependency exists (*A* = 0)." In total, we considered four possible scenarios regarding the age-dependency of DHF (Figure S1 B).

**Simulations**

For each simulation, 100,000 naïve individuals were created. Each individual was assigned randomly to an initial age between 0 and 70 years. Initially, each serotype was introduced into the simulation at a very low inoculation rate (0.0001 per person per time step). The age and immunological state of each individual then were updated at discrete time steps of a half-month, which approximates the incubation and infectious period in a human host [5,19-21]. When an individual reached the 70-year life expectancy, they were replaced with a single new naive subject. Death occurred only by aging, in accordance with the very low level of mortality from DHF at present [22]. We assumed so-called homogeneous mixing as the contact structure: that is, at each time step, an individual was stochastically inoculated with each of the four serotypes according to a probability that was obtained from multiplication of R_0_ and the number of viremic individuals in the preceding time step. The R_0_ varied sinusoidally to mimic seasonality (minimum and maximum were 80% and 120% of average R_0_, respectively). The annual average of R_0_ was kept constant throughout each simulation. When extinction of a virus serotype occurred, the extinct serotype was reintroduced immediately at a very low probability (0.0001 per person per time step), following a widely accepted convention [6]. An individual developed transmissible viremia if he/she was not protected, directly or cross-reactively, to the inoculating virus, regardless of manifesting illness.

Each combination of "*L*", "*C*", "*A*", "*E*" and "*R_0_*" (Table S1) was repeated in eight simulations, and each simulation was run for 150 years, modeling the time elapsed from the emergence of the four serotypes from non-human transmission cycles [23]. We averaged the last 40 years from each 150-year simulation.

The source code of the simulation software can be obtained at http://www.vector-borne-diseases.org/dhf_simulation_plos_ntd. This software can be modified for future studies without notification of the authors.

**Results**

**Effect of cross-immunity ("*C*")**

In accordance with the above mentioned mathematical model, cross-serotype immunity generated a non-monotonic relationship between the incidence of DHF and transmission intensity, R_0_ (Figure S2). This was explained by the fact that, under higher transmission intensity, an increasing number of individuals in the cross-protected state directly entered the totally immune state by bypassing the DHF-predisposed state.

**Effect of age dependency ("*A*")**

We investigated the effects of different age-dependencies in manifesting DHF in secondary infections. As a result, “higher probability of DHF manifestation in older individuals (*A*=2)” and "complex age-dependency (*A*=3)" generated non-monotonic relationships (Figure S3).

**Effect of transmission enhancement ("*E*")**

Transmission enhancement did not result in non-monotonic relationship between incidence of DHF and transmission intensity (Figure S4).

**Temporal pattern of serotype-specific DHF incidence**

The four serotypes exhibited alternating temporal pattern (Figure S5). The periodicity was affected by input parameters, such as duration of cross-protective period, transmission enhancement, and transmission intensity.

**Mean age of DHF cases and dengue transmission intensity**

The mean age of DHF cases was negatively correlated with transmission intensity under any combination of cross-immunity and age-dependency (an example is shown in Figure S6). This result supports the use of a high mean age of DHF cases as a surrogate for low transmission intensity.

**Conclusion**

The individual based model simulation led to non-monotonic relationship between transmission intensity and incidence of DHF in the presence of cross-immunity. This was consistent with the mathematical model on which the individual based model was based. In addition, the individual based model revealed that some forms of age-dependencies in the probability to manifest DHF also can generate such non-monotonic relationship.

**Section II**

**Variation in incidence and duration for averaging.**

In the present study, the variation in incidence of Dengue Hemorrhagic Fever (DHF) at any given value of House Index may seem excessively large. However, the simulations suggested that this level of wide variation could arise from averaging only a three-year duration, even when an ideal measurement of transmission intensity (i.e. basic reproductive number) is available (Figure 6 in the main text). Particularly, simulations that assumed cross-immunity generated a largest variation. Interestingly, the deviance in the predictive model for incidence responded to the duration of window for averaging, in a complex manner both in actual data and in data generated by simulations with cross-immunity (Figure 8 in the main text). We explored explanations for these findings in this section.

**Periodicity and Cross-immunity**

We applied spectrum analysis to the monthly incidence generated from our simulations described in the main text. As a sensitivity analysis, results were compared between diverse durations of cross-immunity, 0, 0.5, 1, 2, 3 and 4 years (Figure S7). Notably, simulations with cross-immunity (≥ two years) resulted in prominent supra-annual periodicities (Figure S7 G – O). This finding is consistent with a previous report that a periodicity of 3-year cycle has been dominant in the incidence of DHF, in Bangkok [24,25]. Especially, within the estimated range of R_0_ values in Bangkok (i.e., R_0_ = 3 – 8 [26]), cross-immunity of two years reproduced this 3-year cycle (Figure S7, G, H). Indeed, there was a remarkable similarity between the spectrum profile generated from the simulation with cross-immunity of 2-year duration (Figure S7 G, H) and that obtained from the actual epidemiological data in Bangkok (Fig. 1 d of ref. [24] ). Therefore, we selected two years as the duration of cross-immunity, in the main text.

We are aware that not only intrinsic (i.e. immunologic) but also extrinsic (i.e. climatic) factors affect periodicity in the incidence of an infectious disease [27]. Since our analysis did not consider the latter, future studies are warranted to disentangle the roles of these factors in the origin of periodicity of DHF.

**Periodicity of Incidence and Variation at Different Windows for Averaging**

Next, we made an attempt to explore how the duration for averaging incidence affected the variation in incidence at a given transmission intensity. We generated a one hundred time-series, in each of which the incidence followed a sinusoidal curve of a fixed amplitude of 100. The baseline of the sinusoidal curve was set to 100. These time-series emulated one hundred districts under the same transmission intensity. The cycle of periodicity ("*T*") was assumed to be (i) one year or (ii) three years. As a result, in *d* th time-series, the incidence at the time-point of *t* (year) would be:

I(*_t, d)_* = 100 + 100 × sin (2π × (*t* + θ*_d_*)/T )

where θ*_d_* denotes the displacement in phase at *d* th time-series. The sinusoidal curves were generated for durations of *W* years (*W*=3, 4, ....40). Annual incidence at *d* th time-series averaged for *W* years was obtained, as in:

Among the hundred time-series, we measured the standard deviation (SD) in M(*_d,w)_*. The resultant SD represents the SD at a given transmission intensity. We randomly selected θ*_d_* at each time-series. Under this assumption of random phases, the SD would respond to *W,* as shown in Figure S8 A. This figure implies that, if *W* is a multiple of *T*, the SD will be 0 regardless the value of *W*; otherwise, the SD will decrease as *W* increases. This finding is explained as follows. If *W* is a multiple of *T*, *W* will contain an equal count of the each phase (e.g., 1st, 2nd and 3rd year for *T*=3), in any time-series. Therefore, the average will be equal in any time-series (i.e., SD=0). On the other hand, if the residue of *W* to base *T* is greater than 0 (for example, residue = 1 for *T*=3), *W* will contain an extra count of 1st phase in some time-series, 2nd phase in other time-series, or 3rd phase in the remaining time-series. This random selection of an extra phase generates variation (i.e., SD>0). However, as *W* increases, the relative contribution of the extra phase to the numerator for averaging will decrease. Therefore, the SD decreases.

Since the actual districts are spatially auto-correlated, the assumption of independent phases is not completely true [25]. To the contrary, if all the time-series are synchronized (i.e., if θ*_d_* is identical for any *d*), the SD would be 0, regardless of *W*. The reality should be between these two extreme assumptions.

Furthermore, we introduced noises to the periodicities, by randomly selecting the cycle at each time-series, between 0.8 and 1.2 years for *T*=1, or between 2.6 and 3.4 years for *T*=3. This generated a more blunt relationship between SD and *W*, as in Figure S8 B. The similarity between Figure S8 B and Figure 8 B in the main manuscript should be noted.

**Conclusion**

Collectively, cross-immunity contributes to generating the supra-annual periodicity in the incidence of DHF. Due to this supra-annual periodicity, which is more dominant than the annual periodicity, the variation in incidence of DHF becomes large and remains high even after the duration for averaging (*W*) is taken considerably long.

**Supplementary Figures**

**Section I**

**Figure S1**. **Individual-Based Model for Dengue Hemorrhagic Fever (DHF)**

A. Diagram of the transition between immunological states caused by infections with wild type virus. The transition between immunological states was a result of either viral inoculation (solid arrow) or expiration of time from the most recent inoculation (broken arrow). The serotype(s) that an individual has experienced is recorded as the existence of protective antibodies to this serotype(s).

B. Age-dependent probability for a secondary infection to manifest as DHF in a DHF-predisposed individual. Four hypothetical possibilities of age-dependency are defined: no age-dependency (*A* = 0), higher probability in younger individuals (*A* = 1), higher probability in older individuals (*A* = 2), and complex age-dependent DHF manifestation (*A* = 3).

**Figure S2. Relationship between DHF incidence and transmission intensity (R_0_) generated by cross-serotype immunity**

Results from simulations, which assumed the cross-protective period ("*C*") to be (A) 0 year, (B) 1 year, or (C) 2 years, are presented. No age-dependency was assumed for DHF manifestation (*i.e.,* *A* = 0). Infection with four serotypes was required to confer life-long resistance to DHF (*L* = 4 serotypes). Transmission enhancement was not assumed (*E*=1). Qualitatively similar results were obtained with *L* = 2 or 3 and with *E* = 2 or 20.

**Figure S3. Relationship between DHF incidence and transmission intensity generated by age-dependent manifestation of DHF**

The results of simulation are presented for four age-dependencies (*A*) of DHF manifestation. No cross-protection was assumed (i.e., *C* = 0 year). Infection with four serotypes was required to confer life-long immunity (*L* = 4 serotypes). Transmission enhancement was not assumed (*E* = 1). Qualitatively similar results were obtained with *L* = 2 or 3, and with *E* = 2 or 20.

**Figure S4. Relationship between DHF incidence and transmission intensity generated by transmission enhancement**

Transmission enhancement (*E*) during manifesting DHF was assumed to be 1 (no enhancement), 2 or 20. No age-dependency or cross-protection was assumed (i.e., *A* = 0, *C* = 0 year). Infection with four serotypes was required to confer life-long immunity (*L* = 4 serotypes). Qualitatively similar results were obtained with *L* = 2 or 3.

**Figure S5. Temporal pattern of alternating serotypes in the presence of cross immunity and effects of a sudden drop in transmission intensity**

Examples of serotype-specific incidence of DHF are presented. The last 40 years are presented.

**Figure S6. Relationship between mean age of DHF cases and Dengue transmission intensity (R_0_)**

Mean age of DHF cases is plotted against transmission intensity (R_0_). The result for a parameter setting (*C* = 2 years, *L* = 4 serotypes, A = no age-dependency, *E* = 1) is presented. All other parameter combinations examined generated similarly negative correlations between mean age of DHF cases and R_0_.

**Section II**

**Figure S7**. Periodicity profile (periodogram) for incidence generated by simulations for Dengue Hemorrhagic Fever (DHF). Individual-based simulation for DHF (described in the accompanying manuscript) was executed for 150 years, from which monthly incidence for the last 40 years was analyzed by fast Fourier transform with Daniell smoothing (provided in R 2.6.2). Parameters for simulations are as follows: cross-immunity of 0.5 year (A – C), one year (D – F), two years (G – I), three years (J – L), and four years (M – O); age-dependency, which attributes a higher probability of manifesting DHF to the older population [defined as *A*=2 in the accompanying manuscript] (P – R); control (i.e., no cross-immunity, no age-dependency) (S – U). Transmission intensity inputted to simulations were R_0_ = 3 (A, D, G, J, M, P, S), R_0_=6 (B, E, H, K, N, Q, T), or R_0_=12 (C, F, I, L, O, R, U). We executed each parameter setting in duplicate, and confirmed that the resulting periodograms were very similar. The highest spectrum intensity was presented as 1, for each parameter setting.

**Figure S8**. Standard deviation in the asynchronous sinusoidal incidences. One hundred sinusoidal curves, with asynchronous phases, were generated, to emulate the incidence of DHF. The sinusoidal incidence was averaged for diverse window lengths ("*W*"), and standard deviation was measured among these averaged incidences. A. Each sinusoidal time-series follows cycles of exactly one year (diamond) or three years (x). B. To add noise to the cycles, the cycle at each time-series was selected randomly between 0.8 and 1.2 years (diamond), or between 2.6 and 3.4 years (x).

**References for Supplementary File**

1. Nagao Y, Koelle K (2008) Decreases in dengue transmission may act to increase the incidence of dengue hemorrhagic fever. Proc Natl Acad Sci U S A 105: 2238-2243.

2. Fisher RA (1930) The general theory of natural selection. Oxford: Clarendon.

3. Macdonald G (1952) The analysis of equilibrium in malaria. Trop Dis Bull 49: 813-829.

4. Brandt WE, McCown JM, Gentry MK, Russell PK (1982) Infection enhancement of dengue type 2 virus in the U-937 human monocyte cell line by antibodies to flavivirus cross-reactive determinants. Infect Immun 36: 1036-1041.

5. Vaughn DW, Green S, Kalayanarooj S, Innis BL, Nimmannitya S, et al. (2000) Dengue viremia titer, antibody response pattern, and virus serotype correlate with disease severity. J Infect Dis 181: 2-9.

6. Ferguson N, Anderson R, Gupta S (1999) The effect of antibody-dependent enhancement on the transmission dynamics and persistence of multiple-strain pathogens. Proc Natl Acad Sci U S A 96: 790-794.

7. Cummings DA, Schwartz IB, Billings L, Shaw LB, Burke DS (2005) Dynamic effects of antibody-dependent enhancement on the fitness of viruses. Proc Natl Acad Sci U S A 102: 15259-15264.

8. Halstead SB (1988) Pathogenesis of dengue: challenges to molecular biology. Science 239: 476-481.

9. Endy TP, Nisalak A, Chunsuttitwat S, Vaughn DW, Green S, et al. (2004) Relationship of preexisting dengue virus (DV) neutralizing antibody levels to viremia and severity of disease in a prospective cohort study of DV infection in Thailand. J Infect Dis 189: 990-1000.

10. Sangkawibha N, Rojanasuphot S, Ahandrik S, Viriyapongse S, Jatanasen S, et al. (1984) Risk factors in dengue shock syndrome: a prospective epidemiologic study in Rayong, Thailand. I. The 1980 outbreak. Am J Epidemiol 120: 653-669.

11. Phuong CX, Nhan NT, Kneen R, Thuy PT, van Thien C, et al. (2004) Clinical diagnosis and assessment of severity of confirmed dengue infections in Vietnamese children: is the world health organization classification system helpful? Am J Trop Med Hyg 70: 172-179.

12. Guzman MG, Kouri G, Valdes L, Bravo J, Alvarez M, et al. (2000) Epidemiologic studies on Dengue in Santiago de Cuba, 1997. Am J Epidemiol 152: 793-799; discussion 804.

13. Guzman MG, Kouri G, Bravo J, Valdes L, Vazquez S, et al. (2002) Effect of age on outcome of secondary dengue 2 infections. Int J Infect Dis 6: 118-124.

14. Ooi EE, Goh KT, Gubler DJ (2006) Dengue prevention and 35 years of vector control in Singapore. Emerg Infect Dis 12: 887-893.

15. Garcia-Rivera EJ, Rigau-Perez JG (2003) Dengue severity in the elderly in Puerto Rico. Rev Panam Salud Publica 13: 362-368.

16. Balmaseda A, Hammond SN, Perez MA, Cuadra R, Solano S, et al. (2005) Short report: assessment of the World Health Organization scheme for classification of dengue severity in Nicaragua. Am J Trop Med Hyg 73: 1059-1062.

17. Lee MS, Hwang KP, Chen TC, Lu PL, Chen TP (2006) Clinical characteristics of dengue and dengue hemorrhagic fever in a medical center of southern Taiwan during the 2002 epidemic. J Microbiol Immunol Infect 39: 121-129.

18. Hammond SN, Balmaseda A, Perez L, Tellez Y, Saborio SI, et al. (2005) Differences in dengue severity in infants, children, and adults in a 3-year hospital-based study in Nicaragua. Am J Trop Med Hyg 73: 1063-1070.

19. Siler JF, Hall MW, Hitchens AP (1926) Dengue: the history, epidemiology, mechanism of transmission, etiology, clinical manifestations, immunity, and prevention. Philippine Journal of Science 29: 1-304.

20. Sabin AB (1952) Research on dengue during World War II. Am J Trop Med Hyg 1: 30-50.

21. Gubler DJ, Suharyono W, Tan R, Abidin M, Sie A (1981) Viraemia in patients with naturally acquired dengue infection. Bull World Health Organ 59: 623-630.

22. Anderson KB, Chunsuttiwat S, Nisalak A, Mammen MP, Libraty DH, et al. (2007) Burden of symptomatic dengue infection in children at primary school in Thailand: a prospective study. Lancet 369: 1452-1459.

23. Wang E, Ni H, Xu R, Barrett AD, Watowich SJ, et al. (2000) Evolutionary relationships of endemic/epidemic and sylvatic dengue viruses. J Virol 74: 3227-3234.

24. Hay SI, Myers MF, Burke DS, Vaughn DW, Endy T, et al. (2000) Etiology of interepidemic periods of mosquito-borne disease. Proc Natl Acad Sci U S A 97: 9335-9339.

25. Cazelles B, Chavez M, McMichael AJ, Hales S (2005) Nonstationary influence of El Nino on the synchronous dengue epidemics in Thailand. PLoS Med 2: e106.

26. Ferguson NM, Donnelly CA, Anderson RM (1999) Transmission dynamics and epidemiology of dengue: insights from age-stratified sero-prevalence surveys. Philos Trans R Soc Lond B Biol Sci 354: 757-768.

27. Pascual M, Cazelles B, Bouma MJ, Chaves LF, Koelle K (2008) Shifting patterns: malaria dynamics and rainfall variability in an African highland. Proc Biol Sci 275: 123-132.
