# Supplementary material for: Relationship between Transmission Intensity and Incidence of Dengue Hemorrhagic Fever in Thailand
Source: PLoS Negl Trop Dis. 2008 Jul 16;2(7):e263. doi: 10.1371/journal.pntd.0000263 (PMC2442222; doi:10.1371/journal.pntd.0000263)
Supplement: Table S1 — Definition and values of scenario parameters assigned to each simulation of Dengue Hemorrhagic Fever (DHF) (0.04 MB DOC) [file pntd.0000263.s002.doc]

**Supplementary Table S1.** Definition and values of scenario parameters assigned to each simulation of Dengue Hemorrhagic Fever (DHF)

| Name | Code | | Parameter range |
| --- | --- | --- | --- |
| (1) Scenario parameters |  |  | |
| (1.1) Age-dependency of DHF manifestation | *A* | 0. No age-dependency.  1. Higher probability of DHF manifestation in younger individuals  2. Higher probability of DHF manifestation in older individuals  3. Complex age-dependency | |
| (1.2) Cross-protective period [years] | *C* | *C* = 0, 0.5, 1, 2, 3, or 4 | |
| (1.3) Number of serotypes sufficient to confer life-long total immunity | *L* | *L* = 2, 3 or 4 | |
| (1.4) Transmission enhancement | *E* | *E =* 1, 2, 20 | |
| (2) Dose parameter |  |  | |
| (2.1) Average basic reproductive number [no dimension] | *R_0_* | R_0_ = 1 – 30 | |
