# Supplementary figures and images for: Relationship between Transmission Intensity and Incidence of Dengue Hemorrhagic Fever in Thailand
Source: PLoS Negl Trop Dis. 2008 Jul 16;2(7):e263. doi: 10.1371/journal.pntd.0000263 (PMC2442222; doi:10.1371/journal.pntd.0000263)

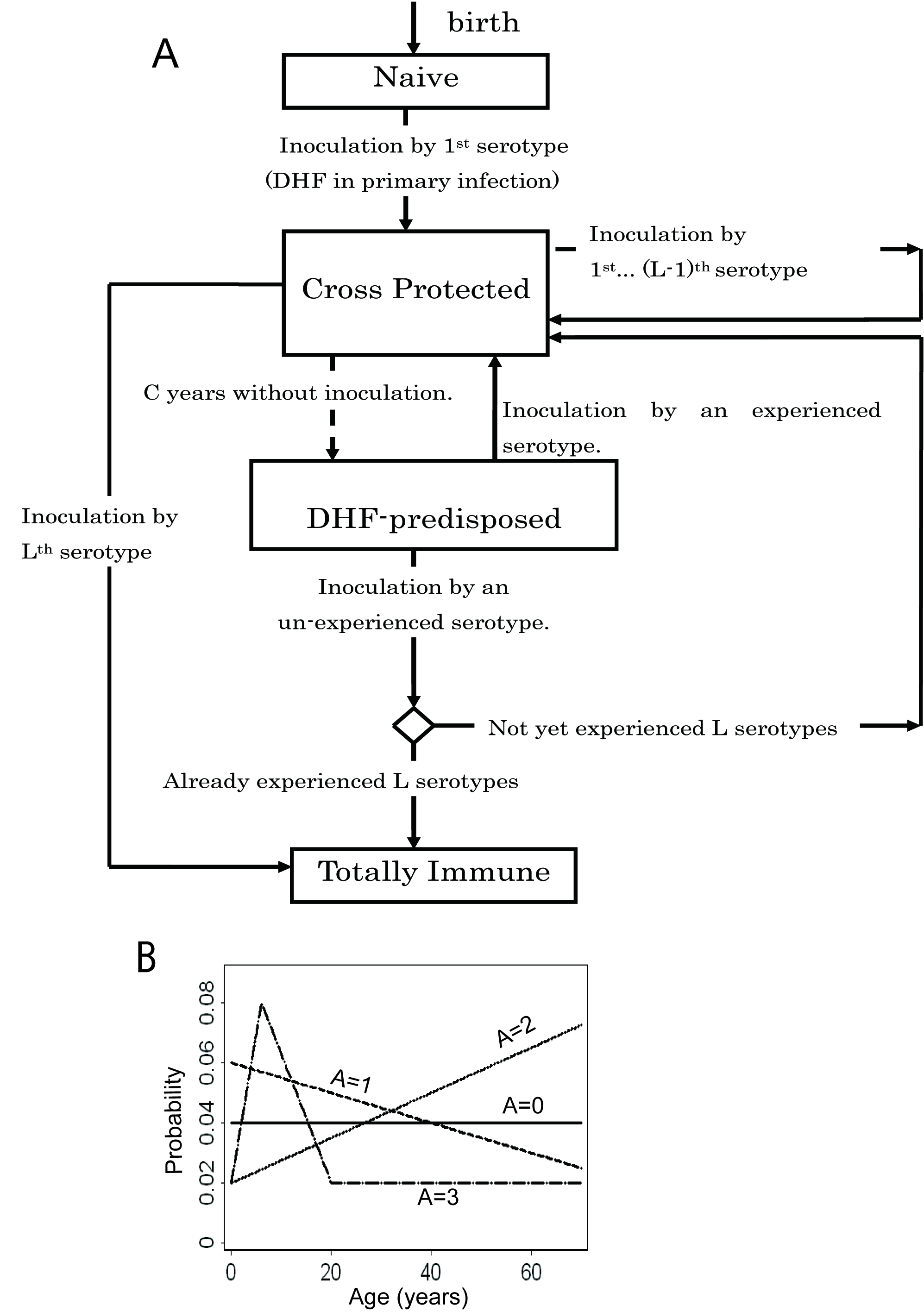

Supplement: Figure S1 — Individual-Based Model for Dengue Hemorrhagic Fever (DHF). A. Diagram of the transition between immunological states caused by infections with wild type virus. The transition between immunological states was a result of either viral inoculation (solid arrow) or expiration of time from the most recent inoculation (broken arrow). The serotype(s) that an individual has experienced is recorded as the existence of protective antibodies to this serotype(s). B. Age-dependent probability for a secondary infection to manifest as DHF in a DHF-predisposed individual. Four hypothetical possibilities of age-dependency are defined: no age-dependency (A = 0), higher probability in younger individuals (A = 1), higher probability in older individuals (A = 2), and complex age-dependent DHF manifestation (A = 3). (0.74 MB TIF) [file pntd.0000263.s003.tif]

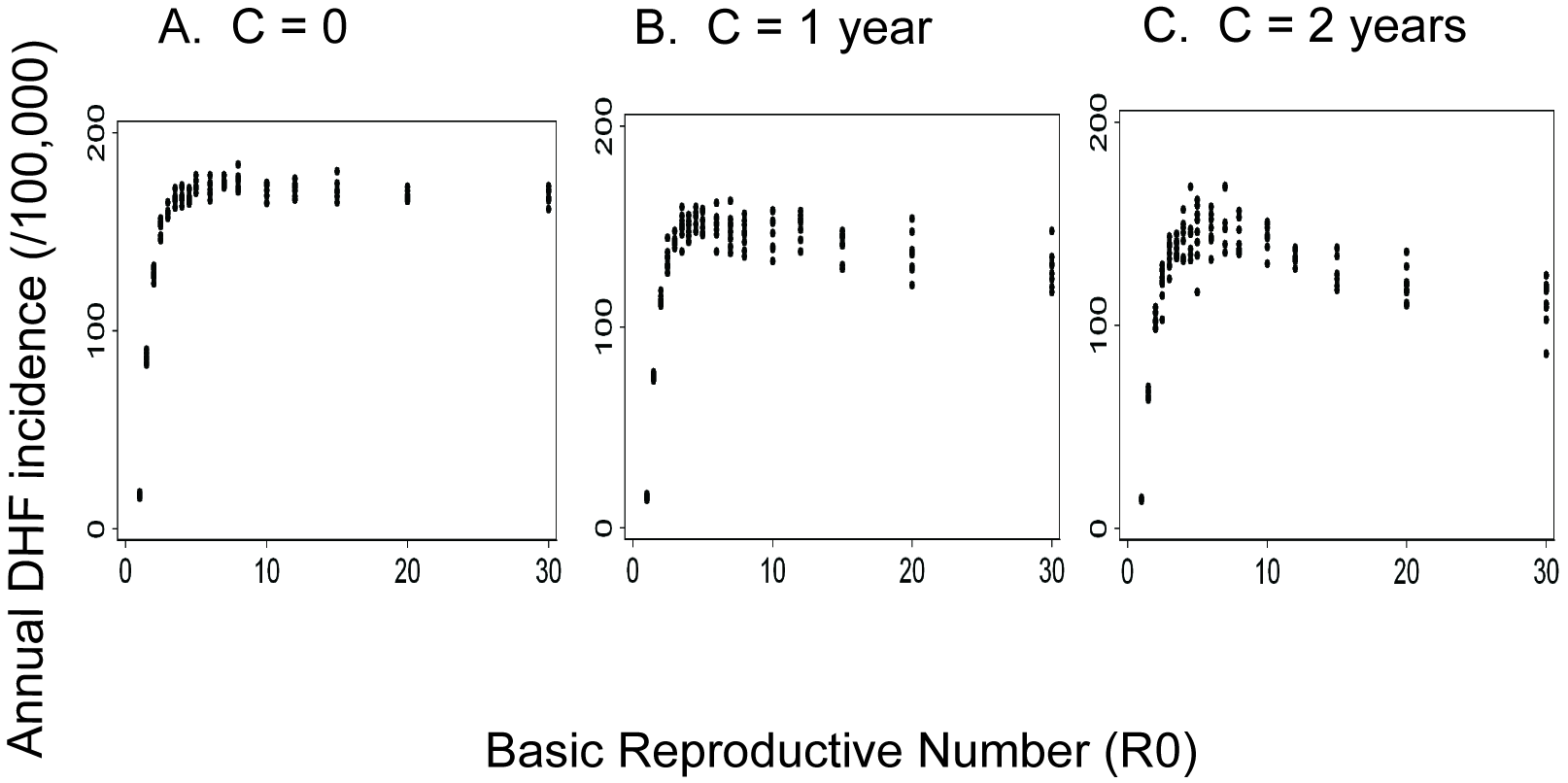

Supplement: Figure S2 — Relationship between DHF incidence and transmission intensity (R0) generated by cross-serotype immunity. Results from simulations, which assumed the cross-protective period (“C”) to be (A) 0 year, (B) 1 year, or (C) 2 years, are presented. No age-dependency was assumed for DHF manifestation (i.e., A = 0). Infection with four serotypes was required to confer life-long resistance to DHF (L = 4 serotypes). Transmission enhancement was not assumed (E = 1). Qualitatively similar results were obtained with L = 2 or 3 and with E = 2 or 20. (0.21 MB TIF) [file pntd.0000263.s004.tif]

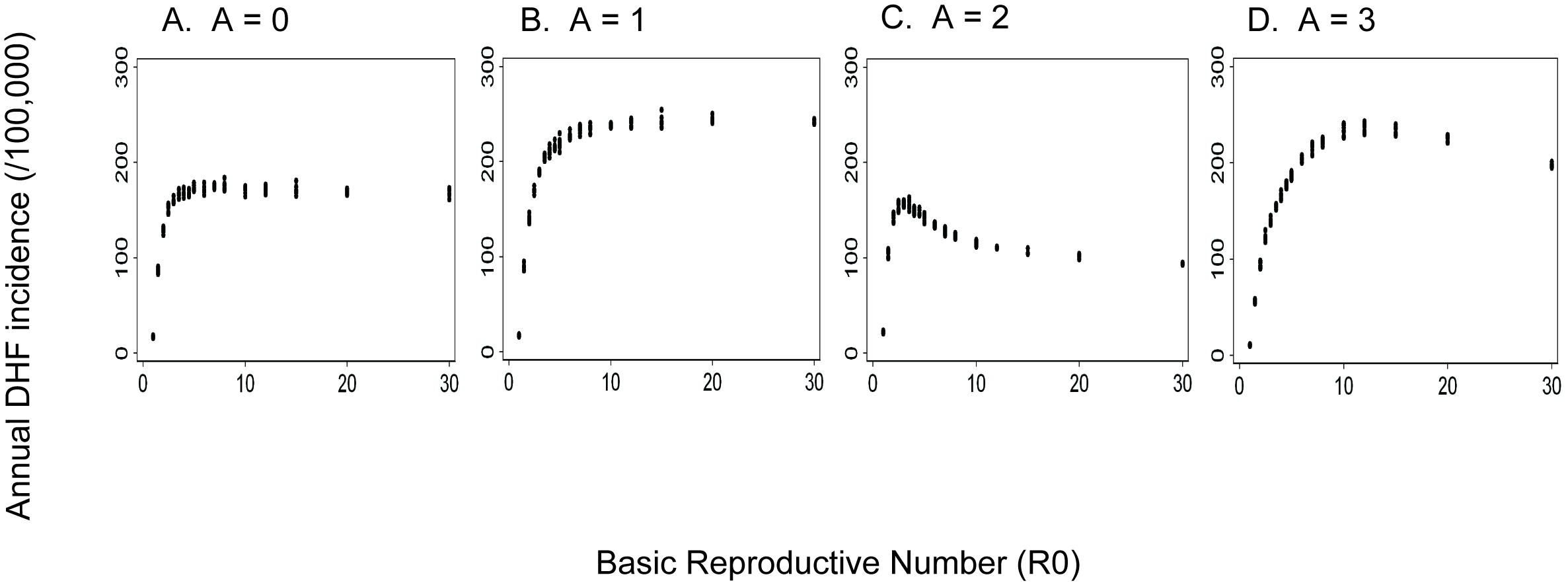

Supplement: Figure S3 — Relationship between DHF incidence and transmission intensity generated by age-dependent manifestation of DHF. The results of simulation are presented for four age-dependencies (A) of DHF manifestation. No cross-protection was assumed (i.e., C = 0 year). Infection with four serotypes was required to confer life-long immunity (L = 4 serotypes). Transmission enhancement was not assumed (E = 1). Qualitatively similar results were obtained with L = 2 or 3, and with E = 2 or 20. (0.26 MB TIF) [file pntd.0000263.s005.tif]

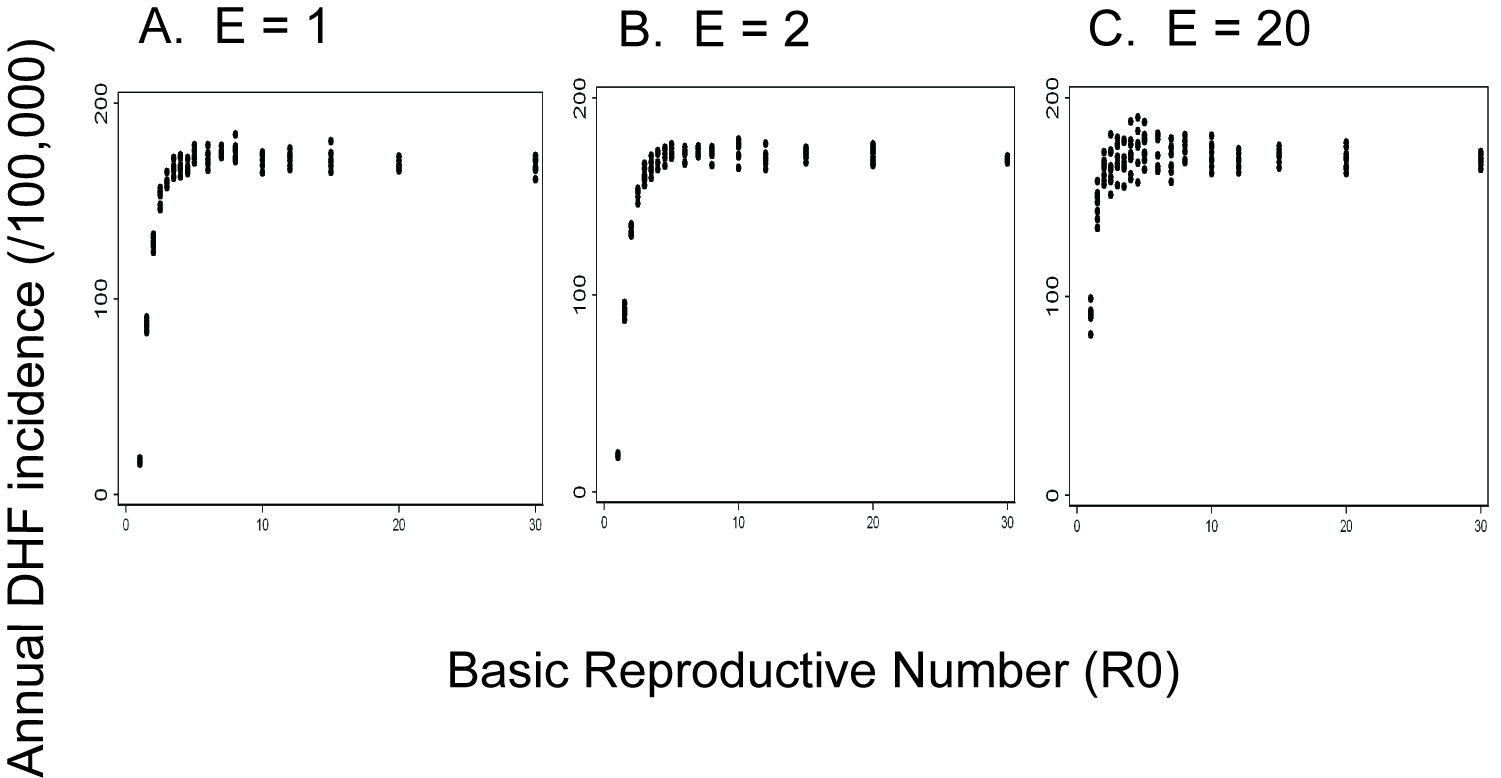

Supplement: Figure S4 — Relationship between DHF incidence and transmission intensity generated by transmission enhancement. Transmission enhancement (E) during manifesting DHF was assumed to be 1 (no enhancement), 2 or 20. No age-dependency or cross-protection was assumed (i.e., A = 0, C = 0 year). Infection with four serotypes was required to confer life-long immunity (L = 4 serotypes). Qualitatively similar results were obtained with L = 2 or 3. (0.18 MB TIF) [file pntd.0000263.s006.tif]

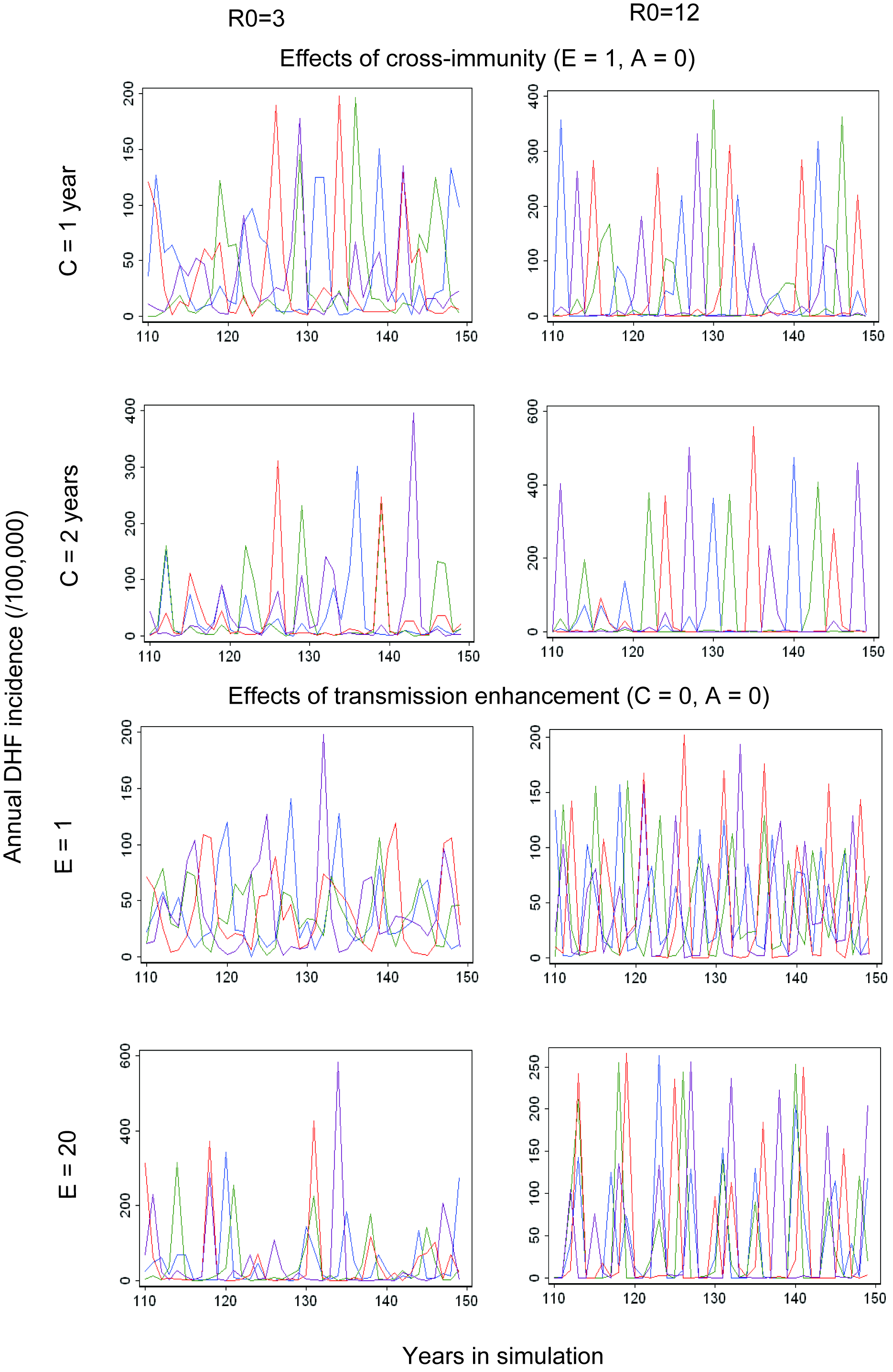

Supplement: Figure S5 — Temporal pattern of alternating serotypes in the presence of cross immunity and effects of a sudden drop in transmission intensity. Examples of serotype-specific incidence of DHF are presented. The last 40 years are presented. (1.18 MB TIF) [file pntd.0000263.s007.tif]

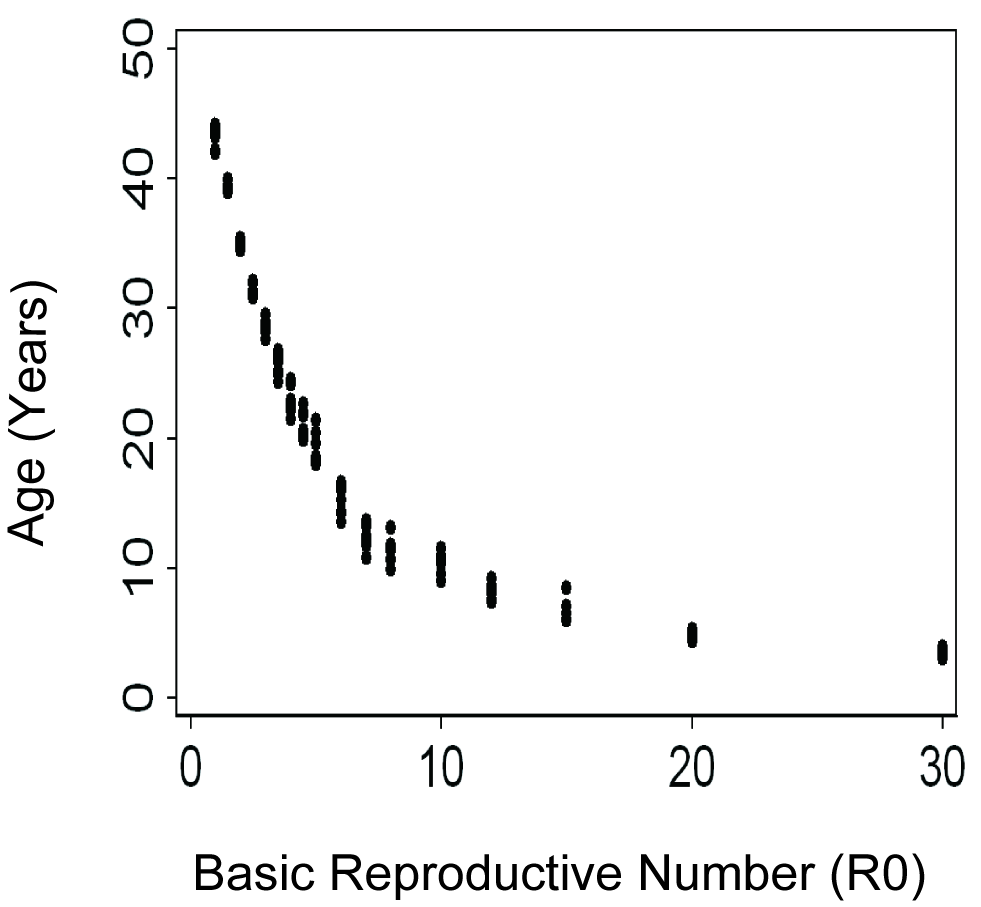

Supplement: Figure S6 — Relationship between mean age of DHF cases and Dengue transmission intensity (R0) Mean age of DHF cases is plotted against transmission intensity (R0). The result for a parameter setting (C = 2 years, L = 4 serotypes, A = no age-dependency, E = 1) is presented. All other parameter combinations examined generated similarly negative correlations between mean age of DHF cases and R0. (0.14 MB TIF) [file pntd.0000263.s008.tif]

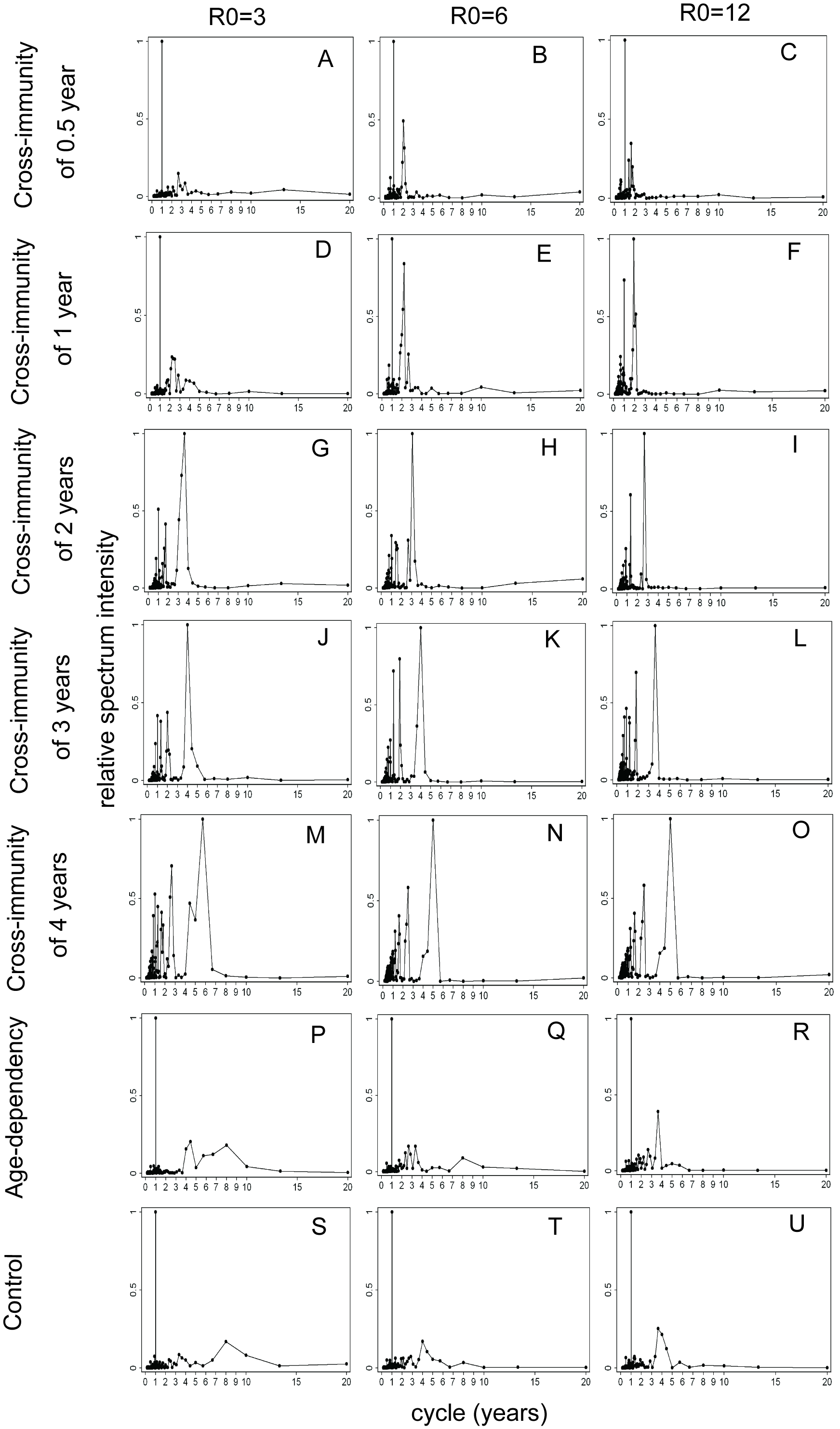

Supplement: Figure S7 — Periodicity profile (periodogram) for incidence generated by simulations for Dengue Hemorrhagic Fever (DHF). Individual-based simulation for DHF (described in the accompanying manuscript) was executed for 150 years, from which monthly incidence for the last 40 years was analyzed by fast Fourier transform with Daniell smoothing (provided in R 2.6.2). Parameters for simulations are as follows: cross-immunity of 0.5 year (A–C), one year (D–F), two years (G–I), three years (J–L), and four years (M–O); age-dependency, which attributes a higher probability of manifesting DHF to the older population [defined as A = 2 in the accompanying manuscript] (P–R); control (i.e., no cross-immunity, no age-dependency) (S–U). Transmission intensity inputted to simulations were R0 = 3 (A, D, G, J, M, P, S), R0 = 6 (B, E, H, K, N, Q, T), or R0 = 12 (C, F, I, L, O, R, U). We executed each parameter setting in duplicate, and confirmed that the resulting periodograms were very similar. The highest spectrum intensity was presented as 1, for each parameter setting. (0.87 MB TIF) [file pntd.0000263.s009.tif]

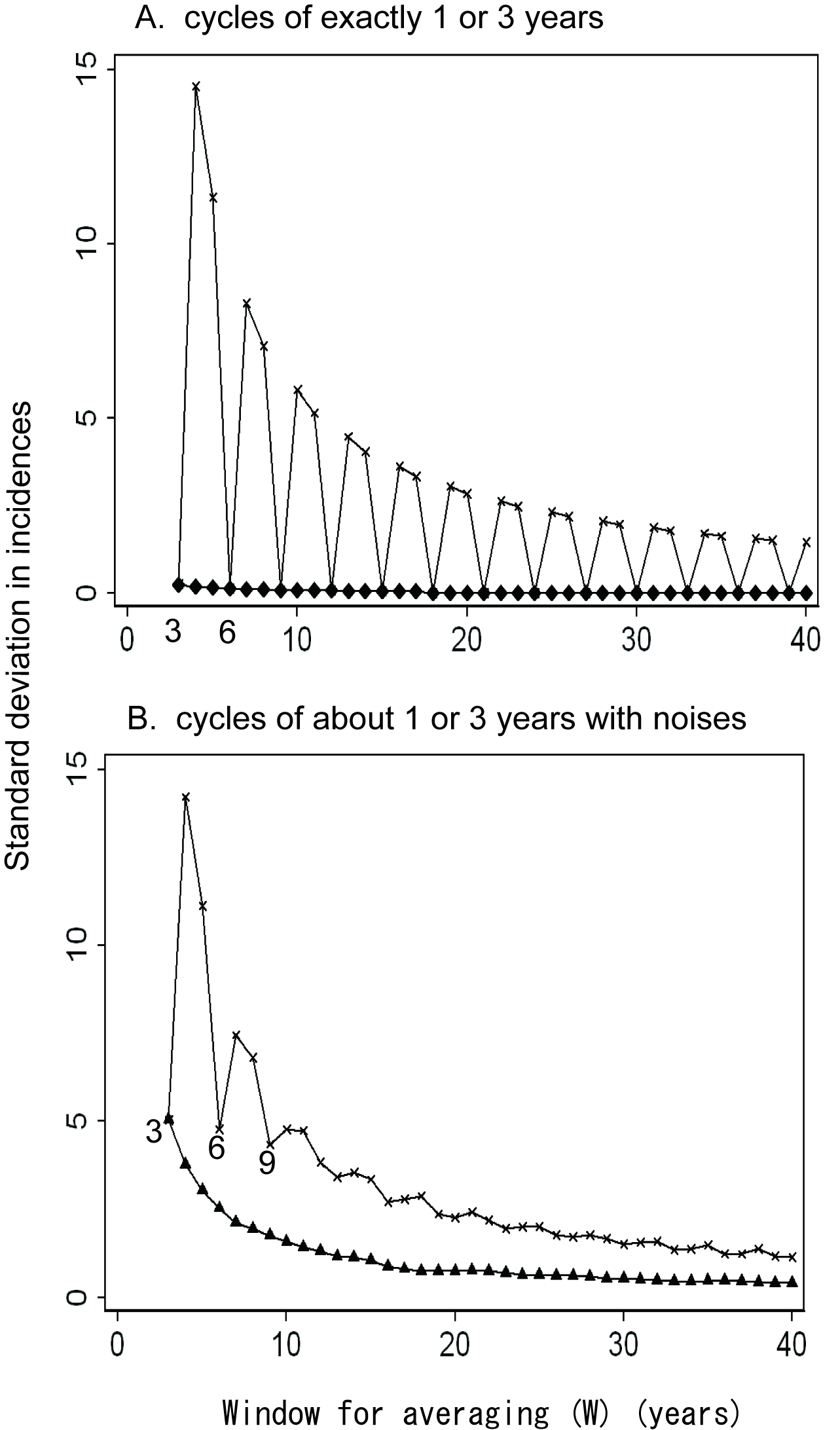

Supplement: Figure S8 — Standard deviation in the asynchronous sinusoidal incidences. One hundred sinusoidal curves, with asynchronous phases, were generated, to emulate the incidence of DHF. The sinusoidal incidence was averaged for diverse window lengths (“W”), and standard deviation was measured among these averaged incidences. A. Each sinusoidal time-series follows cycles of exactly one year (diamond) or three years (x). B. To add noise to the cycles, the cycle at each time-series was selected randomly between 0.8 and 1.2 years (diamond), or between 2.6 and 3.4 years (x). (0.47 MB TIF) [file pntd.0000263.s010.tif]
