# Supplementary material for: Relationship between Transmission Intensity and Incidence of Dengue Hemorrhagic Fever in Thailand
Source: PLoS Negl Trop Dis. 2008 Jul 16;2(7):e263. doi: 10.1371/journal.pntd.0000263 (PMC2442222; doi:10.1371/journal.pntd.0000263)
Supplement: Alternative Language Abstract S1 — Translation of the Abstract into Japanese by Yoshiro Nagao (0.09 MB PDF) [file pntd.0000263.s011.pdf]

背景

デングは蚊が媒介するウィルスとしては感染者数が最も多い。デング出血熱（Dengue Hemorrhagic Fever: DHF）を発症すると死亡する危険もある。DHF は二度目の感染で発症する危険が高い。血清型間に交差免疫が存在するため、デング・ウィルス伝播強度が高い地域において、DHF 罹患率とウィルス伝播強度の間には負の相関関係がある、という仮説が最近、提出された。著者らは、伝播強度を媒介蚊の密度で表すことにより、この仮説を実データに基づいて検証した。

方法と結果

全家屋のうち、媒介蚊のボウフラやサナギが見出される家屋のパーセンテージが House Index (HI)として定義されている。タイにおいて 2002 年から 2004 年にかけて延べ百万軒の家屋を訪問し、HI を求めた。まず始めに、HI と DHF 患者平均年齢が負に相関することから、HI が伝播強度を正しく反映することが確認された。次に、DHF 罹患率と HI の関係を調査した。HI が増加するにつれ、HI が約 30 に達するまでは DHF 罹患率は上昇するが､それ以降は減少に転じた。タイにおいて HI 値が最も高いところから 30 まで HI を減少させると、罹患率は 40%以上増加することが予想された。交差免疫についての上記の仮説に基づいてシミュレーションすると、実データとよく似た結果が得られた。シミュレーションの結果、交差免疫の存在下では、罹患率のバラツキは非常に大きくなり、罹患率と伝播強度の関係は、はっきりしなくなることが予測された。非常に長期間（例えば 10 年以上）のデータを平均してはじめて、この関係は明らかなものとなる。

結論

DHF 罹患率とデング・ウィルス伝播強度が負に相関するということは、高度伝播地域において媒介蚊を中途半端に減らすと、長期的に見て、罹患率が上昇するということを意味する。本研究よりも一層長期間にわたる調査が必要である。
